# Metagenomic time series reveals a Western English Channel viral community dominated by members with strong seasonal signals

**DOI:** 10.1093/ismejo/wrae216

**Published:** 2024-10-23

**Authors:** Luis M Bolaños, Michelle Michelsen, Ben Temperton

**Affiliations:** School of Biosciences, University of Exeter, Exeter, United Kingdom; School of Biosciences, University of Exeter, Exeter, United Kingdom; School of Biosciences, University of Exeter, Exeter, United Kingdom

**Keywords:** viruses, metagenomics, time series, marine microbiology, machine learning clustering, chronotypes, auxiliary metabolic genes

## Abstract

Marine viruses are key players of ocean biogeochemistry, profoundly influencing microbial community ecology and evolution. Despite their importance, few studies have explored continuous inter-seasonal viral metagenomic time series in marine environments. Viral dynamics are complex, influenced by multiple factors such as host population dynamics and environmental conditions. To disentangle the complexity of viral communities, we developed an unsupervised machine learning framework to classify viral contigs into “chronotypes” based on temporal abundance patterns. Analysing an inter-seasonal monthly time series of surface viral metagenomes from the Western English Channel, we identified chronotypes and compared their functional and evolutionary profiles. Results revealed a consistent annual cycle with steep compositional changes from winter to summer and steadier transitions from summer to winter. Seasonal chronotypes were enriched in potential auxiliary metabolic genes of the ferrochelatases and 2OG-Fe(II) oxygenase orthologous groups compared to non-seasonal types. Chronotypes clustered into four groups based on their correlation profiles with environmental parameters, primarily driven by temperature and nutrients. Viral contigs exhibited a rapid turnover of polymorphisms, akin to Red Queen dynamics. However, within seasonal chronotypes, some sequences exhibited annual polymorphism recurrence, suggesting that a fraction of the seasonal viral populations evolve more slowly. Classification into chronotypes revealed viral genomic signatures linked to temporal patterns, likely reflecting metabolic adaptations to environmental fluctuations and host dynamics. This novel framework enables the identification of long-term trends in viral composition, environmental influences on genomic structure, and potential viral interactions.

## Introduction

Viruses are the most abundant entities in the ocean with a vast and diverse genetic functionality [1,2]. Plankton communities are the primary drivers of global biogeochemical cycles [3]. These cycles comprise primary producers and heterotrophic microorganisms that fuel oceanic trophic networks [4], the fluxes of biogenic compounds to and from the atmosphere [5], and their export to the deep sea [6,7]. Therefore, viruses that infect planktonic microorganisms in the ocean play important roles in global biogeochemical cycles by gatekeeping the flux of dissolved organic matter [8,9] or encoding auxiliary metabolic genes (AMGs) to modify host metabolism, impacting processes such as carbon, nitrogen, sulfur, and iron cycling [10–13].

The complexity of the potential outcomes and the environmental forces that control the viral-plankton interactions have been studied in different systems. Examples include the viral enhancement of host fitness in Antarctic bacterial communities during low-nutrient seasons [14], the increase in phytoplankton mortality and viral production in the stratified summer North Atlantic [15], and the influence on shaping the diversity of bacterial ecotypes through space and time [16–18]. Despite these advances, a more detailed ecological and evolutionary understanding of the relationship between viruses, hosts, and environmental conditions is needed to improve our modelling capacity and predict the impacts of warming on oceanic ecosystems [19].

Metagenomics and metatranscriptomics emerged as a cost-effective methodology to analyse viral communities at large scales. Since the retrieval of molecular datasets from the field, it has become evident that viral diversity is immense [20]. Multiple field molecular surveys have expanded our knowledge on the biogeographic distribution of oceanic dsDNA phages [21,22], the abundance and distribution of RNA viruses [23], and the biogeographical patterns of macrodiversity and microdiversity [24]. Field metagenomes have revealed patterns in the genomic population boundaries and the ecological zones in which viral communities can be classified. However, phage-host relationships are shaped in a rapid evolutionary arms race [25], from which transect metagenomes can only offer snapshots of specific time points at different locations.

Oceanographic observatories provide a unique opportunity to overcome this limitation by generating viral metagenomic time series. Temporal patterns of active infections over seasons and sites [26], and diel cycles of viral gene transcription [27,28] have identified a diverse array of activity and life history traits that co-occur between and within viral groups. A handful of continuous viral metagenomics time series have been generated, providing important insights into seasonal succession, phage-host interactions, molecular evolution, and the overall environmental influence on viral composition [29–31]. In different locations and regimes, viral communities show a remarkably stable annual seasonality of populations (viral operational taxonomic units [vOTUs]) in both abundance [32] and composition [29–31,33]. Amid this annual seasonality, the genotypic configuration within populations undergoes constant turnover [29]. This resembles the dynamics outlined in the Red Queen Hypothesis, in which despite a continuous evolution, population fitness remains unchanged [34]. Recently, it has been shown that cyanophages display distinct temporal dynamics driven by the seasonality of the hosts, stochastic environmental processes, and different host range strategies [35]. However, a systematic classification of temporal abundance patterns of a large number of viral genomes or transcripts within a time series remains a major methodological challenge. Generating these classifications represents a milestone in elucidating the associations between temporal abundance profiles of viral populations and specific gene functions, evolutionary patterns, and bottom-up environmental controls.

In this study we generated a monthly viral metagenomic time series of the surface Western English Channel (WEC) to analyse the temporal dynamics of viral populations and the selection of viral genes influenced by seasonal environmental conditions. The WEC is a temperate coastal sea that stratifies seasonally during the summer and is highly dynamic due to the constant riverine input and wind mixing [36]. The WEC microbial community composition has been extensively studied [37–40], providing a perfect framework to analyse virome dynamics. By developing a novel unsupervised machine-learning method, we categorized the temporal abundance patterns of assembled viral contigs from vOTU representative sequences into chronotypes. Chronotypes are statistically discrete clusters of co-varying contigs with similar temporal abundance patterns and optimally separated from other clusters within a dataset. This categorization allowed us to discern between viral populations with specific seasonal and non-seasonal patterns, interrogate whether viral genes are enriched in different temporal patterns, analyse the influence of environmental conditions on chronotypes and evaluate whether distinct evolutionary patterns exist. Our study provides the first detailed insights of viral dynamics in the WEC and their response to environmental variations. Furthermore, we present a novel method of temporal classification that could potentially be applied to analyse other relevant longitudinal molecular datasets, such as amplicon surveys or metatranscriptomes.

## Materials and methods

### Sampling, fractionation, viral DNA extraction, and sequencing

20 L of water samples were collected monthly from the surface (1 m) of the station L4 in the WEC (50°15′ N, 4°13′ W) (Table S1). A total of 27 samples, collected over two and a half years from November 2018 to June 2021, were processed. Concurrent metadata was retrieved from the Western Channel Observatory [41] and the Archive for Seabed Species and Habitats [42]. Chlorophyll *a* concentrations were retrieved from the British Oceanographic Data Centre NOC [43]. Water samples were processed as described previously [44]. Briefly, viral enrichment was done by filtering sequentially the seawater through a glass fiber (GF/D: pore size 2.7 μm) and polyethersulfone (0.22 μm) filters in a 142 mm polycarbonate rig. The viral particles were precipitated using iron chloride and collected on a 1.0 μm polycarbonate filter [45]. Viruses were resuspended in ascorbate-EDTA buffer (0.1 M EDTA, 0.2 M MgCl_2_, 0.2 M ascorbic acid, pH 6.0) using 2 ml of buffer per 1 L of seawater. The resuspended viral fraction was transferred to an Amicon Ultra 100 kDa filter, pre-treated with 1% bovine serum albumin and flushed with SM buffer (0.1 M NaCl; 0.05 M Tris–HCl; 0.8 mM MgCl_2_) [46]. The viral fraction was concentrated to 600 μl and dissolved DNA was digested using DNase I (100 U/ ml; 2 h at room temperature). DNA was extracted using the Wizard DNA Clean-Up System (Promega A7280) and cleaned with a 1.5 × Ampure bead cleanup. 50 ng of DNA were used to construct sequencing libraries in a 1S Plus (Swift Biosciences) library preparation. Libraries were sequenced on a HiSeq 2500 system (Illumina; 2 × 125 bp) to an approximate depth of 25 M reads at the University of Exeter sequencing facility.

### Viral metagenomic processing, assembly, identification, gene annotation, and vOTU definition

All datasets were processed individually following a previously described protocol [24]. Briefly, quality processing was done with BBtools v38.96 [47]. Sequencing adaptors and ΦX174 reads were removed using bbduk (ktrim = r, k = 23, mink = 11, hdist = 1, minlen = 50). Reads that mapped to the human genome were removed using bbmap (minid = 0.95, maxindel = 3, bwr = 0.16, bw = 12, quickmatch, fast, minhits = 2). Short reads were assembled using metaSPAdes from SPAdes v3.15.5 [48]. Contigs with a minimum length of 10 000 bp were used as input to VirSorter v2.2.3 [49]. Contigs of each sample classified as viral were combined and clustered into vOTU (≥95% nucleotide identity and  ≥ 85% coverage) using the Perl script Cluster_genomes_5.1.pl (https://github.com/simroux/ClusterGenomes). The quality of the vOTUs was assessed with CheckV [50]. A final dataset of 3090 high- and medium-quality vOTUs was clustered along with viral genomes from the INPHARED [51] database (July 2023) using vConTACT2 v0.11.1 [52]. Representative contigs of vOTUs were annotated using Pharokka v1.3 [53] and DramV v1.3.5 [54].

### Estimation of vOTU relative abundances

To estimate the relative abundance of vOTUs, quality filtered virome reads were mapped to the representative contigs with bowtie2 v2.3 with default parameters [55]. The generated read alignment files were used to estimate the Reads Per Kilobase per Million mapped reads (RPKM) measurement for each vOTU representative genome of all samples using CoverM v0.6.1 [56] with the following parameters: “--min-read-percent-identity 0.9 --min-covered-fraction 0.4”. A Snakemake (v 5.26.0; [57]) file used to process the viral metagenomes, metadata, and intermediate processing products is available at https://github.com/lbolanos32/WEC_Chronotypes_2024.

### Unsupervised machine learning framework to generate chronotypes

Seasonality, defined as the annual periodic fluctuations of each vOTU, was determined with the Fisher G-test [58] implemented in the GeneCycle package v1.1.5 [59] and based on the RPKMs of the representative contigs. Seasonal and non-seasonal RPKM profiles were analysed independently using a developed clustering method: ChronoClustR (Fig. S1). Briefly, we implemented an iterative unsupervised clustering strategy in which z-score normalized values of the RPKM longitudinal profiles were randomly divided into subgroups of 100 elements. Each of these subgroups was clustered with TSclust v1.3 [60] to calculate the centroids of the optimal number of clusters of each subgroup (defined by having the best Silhouette score) based on the Euclidean distances between them. These centroids were clustered using the K-means method for all potential numbers of clusters (from 1 to n-1 clusters, n = total number of centroids). Based on the curve generated of the relationship between the total within-clusters sum of squares and the number of clusters (*k*), we defined the optimal number of clusters by estimating the closest value of *k* to the first derivative (slope of the tangent line). Three types of clustering (K-means, TSclust, and hierarchical clustering) were generated using the optimal *k* on the original collection. TSclust was selected for this study. These steps were bootstrapped to generate a distribution of the co-occurrence frequency of the vOTU longitudinal profiles in a cluster after multiple rounds of unsupervised clustering. In the current study, we performed 100 bootstrap resamples for both collections of seasonal and non-seasonal vOTUs. A dendrogram was created based on the pairwise co-occurrence matrix of all vOTUs. Finally, the dendrogram was divided using the highest optimal *k* value estimated from all the iterations (see Supplementary methods). The method assumes equidistant sampling time-points with no missing values. Therefore, data points were labelled to the closest equidistant time point within the series and missing values were interpolated using zoo package v1.8 [61]. ChronoClustR is available at https://github.com/lbolanos32/WEC_Chronotypes_2024/tree/main/ChronoClustR.

### Bray–Curtis dissimilarity and environmental Euclidean distances

Averaged Bray–Curtis dissimilarities were used to generate time-decay curves [62,63]. These were generated for both seasonal and non-seasonal subsets. To identify whether the seasonal signal was driven by the most abundant vOTUs, we further divided seasonal and non-seasonal fractions into the first quartile, interquartile range, and third quartile of the RPKM distribution. A harmonic linear sinusoidal regression was generated in R v4.3 [64] and evaluated to confirm a significant seasonal amplitude in the data. The values of the environmental variables were standardized on a 0 to 1 interval, based on the minimum and maximum values of each variable, using the formula: ${X}^{\prime }=\frac{x-\min (x)}{\max (x)-\min (x)}$. Euclidean distances between consecutive months were estimated using these normalized values. Wavelet transforms of Bray–Curtis dissimilarities and environmental Euclidean distances of subsequent samples, and the wavelet coherence analysis of these were performed with WaveletComp [65] following previously described methods [66].

### Differential gene abundances and medoid correlations with environmental parameters

To assess the differential gene abundances between seasonal and non-seasonal chronotypes, we used the normalized values obtained by dividing the gene frequency by the number of members within a chronotype. One-tailed Student’s t-tests with Bonferroni correction for multiple testing were performed in R v4.3 [64] for all the PHROG category ratios (gene count/chronotype members). Spearman pairwise correlations were calculated for all combinations of chronotype medoids and environmental parameters. The corr.test function in Psych v2.3.9 [67] was used to report the probability values of each pairwise comparison (Holm method for adjustment of multiple tests).

### Phylogenetic analysis

Predicted proteins belonging to significant differentially abundant gene categories between seasonal and non-seasonal chronotypes were analysed. To determine whether the enrichment of potential AMGs reflects a particular adaptation of a majority of seasonal pelagiphages (enriched in co-occurring ferrochelatase and 2-OG(FeII) oxygenases) [68] or a broader observation, we analysed together both the vOTU proteins and those annotated from known pelagiphage genomes. Genomes from cultured and curated metagenome assembled pelagiphages [68–73] were re-annotated as previously described to standardize functional prediction. Predicted proteins annotated as ferrochelatase, primase, or 2-OG(FeII) oxygenases were retrieved and aligned together using t-coffee v12.0 [74]. Phylogenetic trees were constructed using IQ-Tree v2.2 [75] with the following parameters: “-msub viral -bb 1000 -mset”. The best model selected for each category was: rtREV+F + R5 for ferrochelatase, rtREV+F + R7 for primase, and rtREV+F + I + R9 for 2-OG(FeII) oxygenases. Newick files were processed in R using ape v5.7 [76] and plotted with ggtree v3.8 [77].

### Polymorphism profiles

Single nucleotide polymorphisms were called as previously described [29]. Briefly, variation per site was estimated using samtools [78] and bcftools [79] on a subset of completely aligned reads with a minimum identity of 98% with the following parameters: “mpileup -g -f | bcftools call -ploidy 1 -mv”. Variants were filtered using “filter -e ‘%QUAL<20 || DP<10”; with a frequency > 1%. The temporal variation profiles for 26 851 vOTUs longer than 10 000 bp were generated by counting shared polymorphisms between dates. No cross-assembly was performed to avoid potential chimeras and keep a consistent dataset of vOTUs. Two analyses were done (a) considering only those vOTUs that had a minimum coverage of 10× across 90% of its length throughout all the samples [29] and (b) considering vOTUs that had a minimum coverage of 10× across 90% in at least one sample. Only 56 (out of the 3090 high-quality) vOTUs fulfilled these requirements in the analysis (a). These vOTUs represent a ubiquitous and abundant collection of viral sequences in the time series. 2997 out of the 3090 high-quality vOTUs fulfilled the conditions for analysis (b). Variant density was calculated for both sets as follows: $Variant\ density=\frac{variants}{\left( vOTU\ length\right)\ast (coverage)}$. SNP profiles were compared using custom bash scripts. Figures were plotted using ggplot2 v2.3 package [80]. Figures were edited in Inkscape2 (www.inkscape.org) for aesthetics. R and bash scripts to generate the figures of this manuscript are available at https://github.com/lbolanos32/WEC_Chronotypes_2024.

## Results

### Western English Channel vOTU composition displays a consistent annual cycle with seasonal variation in rates of change

**Figure 1:**
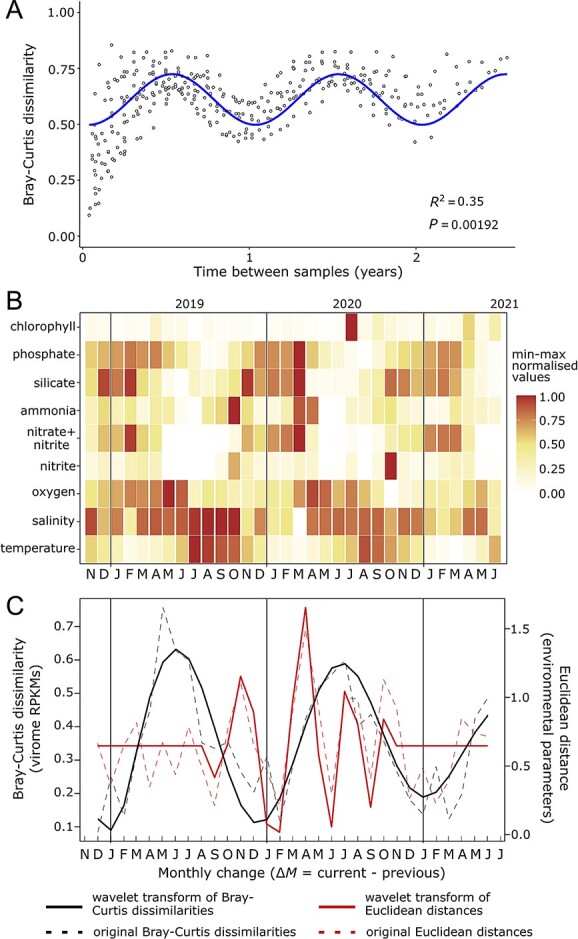
**Bray–Curtis dissimilarity time decay analysis of the virome composition in the surface Western English Channel and its relationship with environmental changes.** Pairwise Bray–Curtis dissimilarity in the viral community was estimated using the reads per kilobase per million mapped (RPKMs) of 3090 representative contigs over a period of more than two and a half years. (**A**) Bray–Curtis dissimilarity time decay analysis. Bray–Curtis dissimilarities were averaged to establish a correlation with time distance (time gap between samples). A harmonic linear regression model was employed to identify significant seasonal trends in both the complete dataset and the analysed fractions. A harmonic linear regression model was employed to identify the significant seasonal trend (*P* < 0.05). (**B**) Normalized values (0–1) of the nine environmental parameters used to estimate the Euclidean distance between monthly consecutive samples. (**C**) Wavelet transforms (solid red line) of the monthly consecutive Euclidean distances representing the environmental changes and Bray–Curtis dissimilarities of the viral communities (solid black line). The original Euclidean distances and Bray–Curtis dissimilarities are shown as dashed lines, red and black respectively. The one-letter monthly notation on the x-axis represents the difference between the labelled month and the previous month (e.g. “D” represents the difference between December and November). Changes in Euclidean distances and Bray–Curtis dissimilarities did not show a significant (*P* < 0.01) in-phase oscillation (Fig. S4).

A total of 26 851 vOTUs were generated from 27 monthly surface metagenomes spanning more than two and a half years in the WEC. Out of these, 3090 (11.5%) were classified as high-quality viral assembled contigs. Only nine (0.3%) of these high-quality vOTUs were classified as ssDNA phage. We analysed the seasonal dynamics of the overall communities represented by these 3090 vOTU contigs throughout the time series. The Bray–Curtis dissimilarity index of all the possible pairs of samples was estimated and the average plotted as a function of the temporal distance between them (Fig. 1A). The significant sinusoidal pattern revealed peaks of dissimilarity at the 6-, 18-, and 32-month time gaps, while communities exhibit a maximum similarity at 0-, 12-, and 24-month time gaps between them.

To interrogate whether this strong seasonal signal was driven by a few abundant contigs, we fractionated the high-quality populations based on the distribution of the calculated RPKMs. The first quartile, the interquartile interval and the third quartile were extracted, and Bray–Curtis dissimilarities were recalculated. The three fractions exhibited similarly remarkable annual stability, suggesting that seasonality is not dependent on abundance. Using the Fisher G-test for time series, 2484 contigs (80.4%) were categorized as seasonal, and 606 (19.6%) as non-seasonal (Fig. S2). A sinusoidal harmonic linear regression was fitted to each of these categories to confirm that the signal was significantly correlated with the seasonal fraction Bray–Curtis dissimilarities. The Bray–Curtis dissimilarities between the seasonal contigs displayed a significant correlation to the sinusoidal regression, which was absent in the non-seasonal fraction (Fig. S2). Similarly, each of these groups was divided into the three quartiles described before. In the seasonal fraction, the seasonality signal was present in all quartile ranges evaluated, while absent in each of the non-seasonal quartiles. These temporal patterns were supported when the extended 26 851 vOTUs dataset was analysed (Fig. S3). A residual seasonal signal might be observed in the non-seasonal quartiles because of the influence of weaker periodic signals that did not meet the Fisher Gtest significance threshold. These results suggest that viral communities are mostly composed of prevalent seasonal populations with a wide distribution of abundances, and a smaller fraction displaying irregular non-seasonal temporal patterns.

We evaluated whether monthly environmental changes influence the rate of change of the virome composition. Consecutive Euclidean distances were generated based on nine environmental measurements (Fig. 1B). Wavelet transforms of the monthly consecutive Euclidean distances and Bray–Curtis virome composition dissimilarity were estimated and compared. Bray–Curtis virome dissimilarity showed an oscillatory trend in which the rate of change increases sharply from December–January to June–July. Meanwhile, this rate decreases from June–July to December–January (Fig. 1C). Euclidean distances showed a variable pattern of changes between months. No significant common oscillatory behavior (*P* < 0.01) was found between Euclidean and Bray–Curtis distances during the studied period (Fig. S4).

### Viral populations that show similar patterns of longitudinal abundance can be clustered into highly defined chronotypes

Among the seasonal and non-seasonal groups of viral populations, different contigs exhibit comparable temporal patterns. To have a highly defined characterization of these patterns, we developed and employed a novel unsupervised machine learning method, denominated ChronoClustR, to de novo cluster them into chronotypes. A chronotype is defined as a cluster of viral population representatives that have similar longitudinal abundance patterns over time and are optimally maximized to be different enough from other temporal clusters within the community.

**Figure 2:**
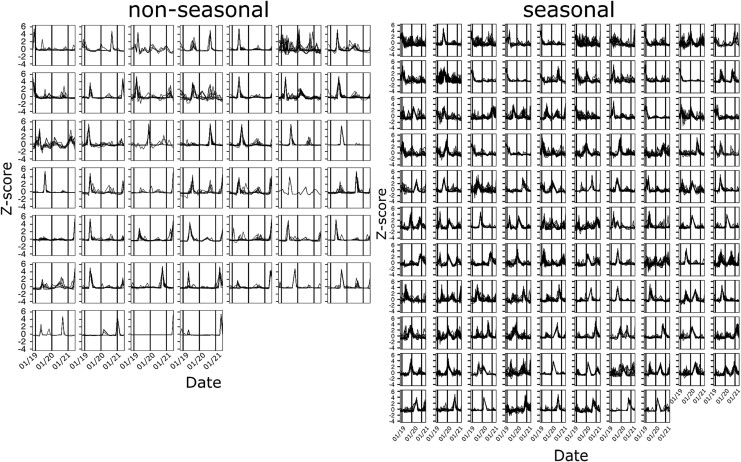
**Seasonal and non-seasonal chronotypes derived from an unsupervised machine learning clustering method.** Z-score normalized values of each of the 3090 longitudinal RPKMs organized and plotted by its chronotype membership, represented by each panel. On the left side, 606 non-seasonal normalized RPKMs time series are represented in 46 different chronotypes. On the right side, 2484 seasonal normalized RPKMs time series are represented in 108 chronotypes.

Among the 2484 seasonal contigs, 108 chronotypes were determined using ChronoClustR, while 46 chronotypes were determined from the 606 non-seasonal contigs (Fig. 2). The optimal number is the maximum *k* determined throughout the bootstrap replicates. Based on vConTACT2 taxonomic characterization, viral genera diversity increased linearly with the number of members in a chronotype, in both seasonal and non-seasonal chronotypes, without reaching a plateau (Fig. S5). 26% seasonal and 22.5% of non-seasonal contigs were clustered with at least one representative genome with a known host (Fig. S6a). From these, the top three differences between seasonal and non-seasonal relative abundances included pelagiphages and methylophages, which were 2.1 and 3 times more represented in the seasonal fraction, respectively, while Nonlabens (marine Flavobacteria) phages were 2.6 times more represented in the non-seasonal fraction (Fig. S6b).

### Seasonal chronotypes show a significant enrichment in 2-OG(FeII) oxygenases and ferrochelatases, potential AMGs

**Figure 3:**
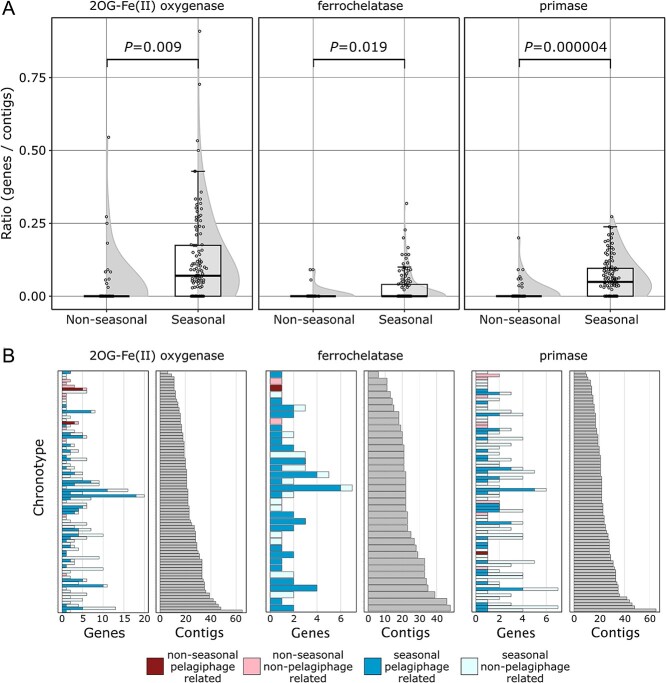
**Seasonal chronotypes have a significant higher number of 2-OG(FeII) oxygenases, potential auxiliary metabolic genes (AMGs).** (**A**) Distribution of normalized gene observations in seasonal and non-seasonal chronotypes of the significantly different functional categories. Violin plots and box plots are overlapped to show the distribution of the chronotype gene/contig ratios. Gene categories are organized from left to right: 2-OG(FeII) oxygenase (phrog61), ferrochelatase (phrog2954), and primase (phrog21866). (**B**) Breakdown of gene origins for each chronotype with at least one occurrence for the three seasonal enriched categories (left panels). Stacked bar plots are color-coded based on the seasonality pattern of each chronotype and arranged vertically by the number of contigs, from the least to the highest number of members (top to bottom). Darker colors within the bar plots represent genes derived from contigs taxonomically related to pelagiphages and lighter colors indicate other origins. The number of contigs for each chronotype is displayed as an aligned bar plot (right panels).

We hypothesized that characteristic AMGs from vOTUs contigs associated with strongly seasonal hosts at the WEC (i.e. *Synechococcus*) [40], such as cyanophage-encoded photosystem ll genes, would be enriched in seasonal chronotypes [81]. Out of the total 379 PHROG functional categories detected in all vOTU contigs, three showed a significant differential (*P* < 0.05) abundance between those in seasonal versus non-seasonal chronotypes: 2-OG(FeII) oxygenase superfamily, ferrochelatase, and primase (Fig. 3A). 2-OG(FeII) oxygenase and ferrochelatase genes have been found to co-occur in pelagiphages genomes [68] and catalyze the oxidation of multiple compounds [82]. 2-OG(FeII) oxygenases and ferrochelatases co-occur in 33.5% seasonal contigs with at least one 2-OG(FeII) oxygenase gene (Fig. S7) and 70% of this fraction are pelagiphage related (Fig. S6c). These are defined as contigs that clustered with at least one known pelagiphage genome based on a protein cluster-based gene sharing network (vConTACT2), taxonomically placing them in the same genus. Most of the genes from these three categories were retrieved from seasonal pelagiphage related and uncharacterized contigs (Fig. S6c). Specifically, 40.1% of seasonal 2-OG(FeII) oxygenases, 70.6% of ferrochelatases, and 30% of primases were derived from pelagiphage-related contigs, with representation on 46.6%, 75.7%, and 43.3%, respectively, of the total seasonal chronotypes with at least one occurrence (Fig. 3B). Contrary to our hypothesis, AMGs enriched in seasonal chronotypes did not include those associated with cyanophage photosystem manipulation. Cyanophage related contigs represented only the 1.04% of the total seasonal contigs (Fig. S6b) and just two photosystem II D1 genes were retrieved from these contigs.

**Figure 4:**
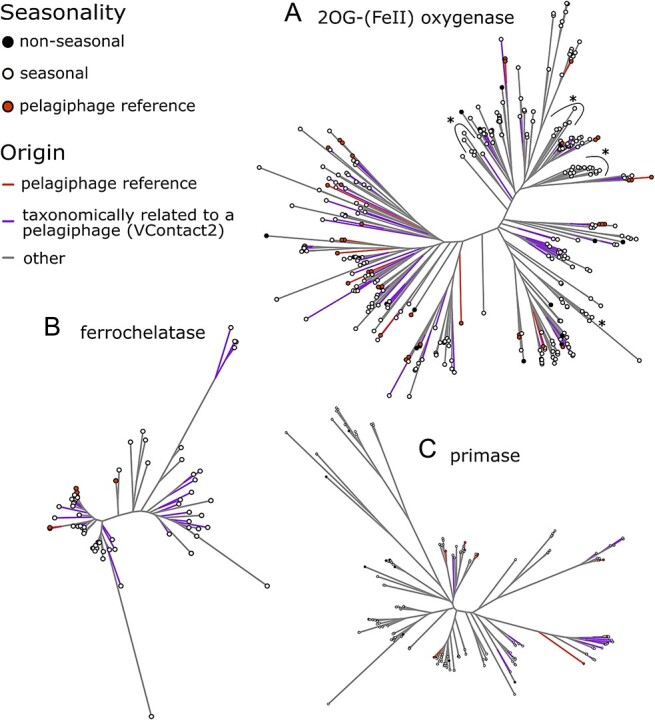
**Phylogenetic analysis of the seasonal enriched gene categories.** Maximum likelihood phylogenetic trees of the translated coding sequences of the genes driving the differences between seasonal and non-seasonal chronotypes. For all the phylogenetic trees, branches are color-coded based on the vOTU where the predicted proteins were originated: red for the pelagiphage references, purple for vOTUs clustered with pelagiphage genomes (taxonomically related to pelagiphages at the genus level), and gray for other (non-pelagiphage taxonomically related vConTACT2 cluster). The terminal nodes are overlapped with a color-coded circle indicating the temporal pattern, seasonal vOTU or non-seasonal vOTU, of the genome which the coding sequence was retrieved or if it was retrieved from known pelagiphage genomes. Asterisks indicate clades without any taxonomically related pelagiphage origin protein. (**A**) 2OG-Fe(II) oxygenase (**B**) primase, and (**C**) ferrochelatase.

To further investigate the relationship between the seasonality of 2-OG(FeII) oxygenases, ferrochelatases, and primases, their taxonomy, and phylogenetic relationships, we performed a maximum likelihood phylogenetic analysis of all the translated proteins of the coding sequences (Fig. 4). These gene functional categories have representatives in cultivated pelagiphage genomes, which allow us to accurately evaluate whether these genes occur broadly through the viral diversity or are a constrained genomic feature of abundant and seasonal pelagiphages. The 2-OG(FeII) oxygenase phylogenetic tree shows multiple lineages that might be related to the functional diversification of this family (Fig. 4A). 2-OG(FeII) oxygenases from cultured pelagiphages and the metagenomic proteins derived from pelagiphage related vOTUs are distributed throughout most of the phylogenetic tree clades. Similarly, ferrochelatases are broadly distributed in the phylogenetic tree (Fig. 4B). In contrast, primases related taxonomically to pelagiphages are constrained mostly to three clades, while those derived from the reference genomes are scattered throughout the tree. (Fig. 4C). The gene-sharing profile classification of metagenomic assembled contigs with pelagiphages by vConTACT2 is not intended to predict the host of vOTU contigs, but to provide taxonomical context of the contigs containing these sequences. Therefore, we can interpret that beyond the host, the expanded seasonal taxonomical groups related to the analysed pelagiphages are enriched with the three described gene categories.

The enrichment of seasonal chronotypes with 2-OG(FeII) oxygenases and co-occurring ferrochelatases (Figs. 3,4,S6,S7) underscores the viral adaptations and their influence on the seasonal cycle of their hosts by potentially expanding and manipulating their metabolic capacity. Furthermore, no specific AMGs were found that could broadly explain the differences between seasonal and non-seasonal vOTUs. Instead, the enriched categories appeared to be mostly associated with highly represented seasonal pelagiphage-related contigs (Figs. 3B,S6c).

### Chronotypes are primarily influenced by temperature and nutrient concentrations

**Figure 5:**
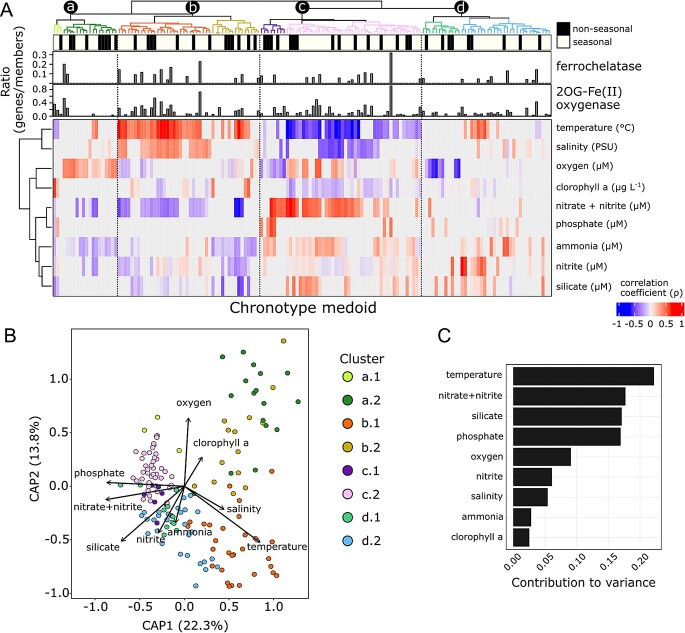
**Environmental correlation analysis with medoid representatives of the chronotypes.** (**A**) Correlation heatmap between chronotype medoids and oceanographic environmental parameters. The correlation values are color-coded (blue to red) if the correlation was significative (*P* < 0.05). The top dendrogram represent the medoid clustering based on the correlation profiles of the heatmap. The terminal node attached bar plot indicates the temporal pattern, seasonal or non-seasonal, of the medoid. Below, two bar plot panels depict the distribution of the auxiliary metabolic genes, ferrochelatase, and 2OG-Fe(II) oxygenase, in the chronotype represented by the medoid. Based on the correlation profile, four groups of chronotypes are distinguished. “a” medoids mostly correlate positive with oxygen concentration. “b” medoids positively correlate with temperature and salinity, while negatively with nutrients (NO_2_ + NO_3_, PO_4_, SiO_2_). “c” medoids mostly correlate positively with nutrients (NO_2_ + NO_3_, PO_4_, SiO_2_, NH_4_), while negatively correlated with temperature and salinity. “d” medoids do not show a consistent pattern of correlations. (**B**) Canonical analysis of principle coordinates (CAP) of medoid temporal profiles constrained by the measured environmental parameters. Bray–Curtis dissimilarities were estimated using the derived medoid RPKM values. Axes depict the first (x) and second (y) constrained components. Arrows represent the explanatory variables and their length its contribution to the explained variation. The orientation of the arrow represents the direction to which the variable increases. Medoids are color coded based on its membership to the subgroups from the clustering of panel (a). (**C**) Bar plots depicting the overall contribution to the variance of each environmental variable.

To evaluate the potential effects and influence of environmental conditions on the temporal patterns of seasonal and non-seasonal chronotypes, we identified the medoids for each chronotype and generated correlation profiles with the available surface water physico-chemical variables (Fig. 5A). Based on the correlation profiles, two major groups were identified and separated by differential correlations with temperature, salinity, and nutrients. These two large trends are represented by clade b and c. Clade b chronotypes are positively correlated with temperature and salinity, while negatively correlated with nutrients (NO_2_ + NO_3_, PO_4_, SiO_2_). Clade c chronotypes are negatively correlated with temperature and salinity and positively with those nutrients. The presence of 2-OG(FeII) oxygenases and co-occurring ferrochelatase AMGs did not show an enrichment pattern in any of the previously described clades but were consistently found in seasonal chronotypes regardless of their relationship with environmental variables. While the identified chronotype groups exhibit varied correlations with temperature, dissolved oxygen, salinity, and nutrients, the absence of a consistent correlation of the seasonal-enriched AMGs with the evaluated parameters suggests that the functional diversity of these genes allow them to be selected within multiple environmental conditions. A constrained ordination and the variable contribution to the variance analysis support that temperature is the most important driver of the medoid temporal patterns, followed by the dissolved nutrient concentration of NO_2_ + NO_3_, SiO_2_, and PO_4_ (Figs. 5B,5C).

### Viral contigs undergo rapid polymorphism turnover, with a few seasonal chronotype members displaying annual recurrence of genetic variants

**Figure 6:**
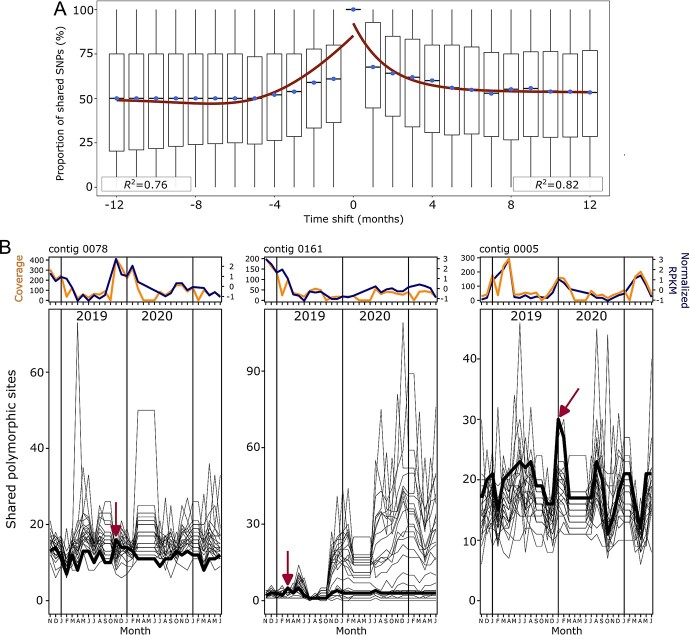
**Temporal dynamics of polymorphic profiles of ubiquitous and abundant vOTUs.** (**A**) Comparison of the polymorphic profiles of the 56 prevalent and abundant vOTUs (minimum coverage of 10× across 90% of the length throughout all samples). A model incorporating exponential growth coupled with a sigmoid transition based on the median values of the distribution over time was generated. A red line representing the time-shift values under the model is overlaid on the original median values. The R-squared value indicates the goodness of fit of the exponential-sigmoid model to the median values of each segment. (**B**) Three examples of individual shared polymorphic profiles from the collection of 56 vOTUs. Abundance pattern is shown on the top panels by (i) coverage (in yellow and fixed in the left y-axis) and (ii) z-score normalized RPKM (in blue and fixed in the right y-axis). A pairwise comparison of shared polymorphic sites is shown in the bottom panels. Each line represents the collection of comparisons of a fixed month to the rest of the profiles. A red arrow indicates the sample of origin of the representative vOTU sequence. The polymorphic profile time series of the population where the contig was retrieved has been highlighted with a black bold line to exemplify different patterns of succession. Left panel vOTU: The highlighted polymorphic dynamics from November 2019 profile have a similar number of shared polymorphic sites 12 months before (November 2018). Mid panel vOTU: The highlighted polymorphic dynamics remained mostly constant and with low variation compared to the fixed profile of March 2019. Right panel vOTU: The highlighted polymorphic dynamics show an increase towards the fixed profile of January 2020 and a later overall decrease with recurrent upticks.

We sought to test whether the Red Queen dynamics shown in a relatively stable system such as the San Pedro Ocean Time series (SPOT) [29] would be evident in the WEC, which undergoes greater fluctuations in mixing and temperature over both short and long timescales [66]. Intra-population genotypic changes throughout the time series were analysed by comparing the genetic polymorphic profiles of each vOTU. First, we analysed the polymorphic profiles of those vOTUs (>10 kb) with a minimum coverage of 10× across 90% of its length throughout all the samples. This dataset was composed of 56 high quality vOTUs (4472 SNPs). Pairwise comparison of population profiles at each month compared with previous and subsequent months showed that the genetic similarity, represented by the percentage of shared polymorphisms, decreases over time (Fig. 6). The median of the distribution of the decrease on shared polymorphisms stabilizes at 50% before and after five months from a fixed profile (Fig. 6A). Individual vOTUs analysis reveal multiple patterns of rise and decay with variable recurrences, rather than linear trends (Fig. 6B).

**Figure 7:**
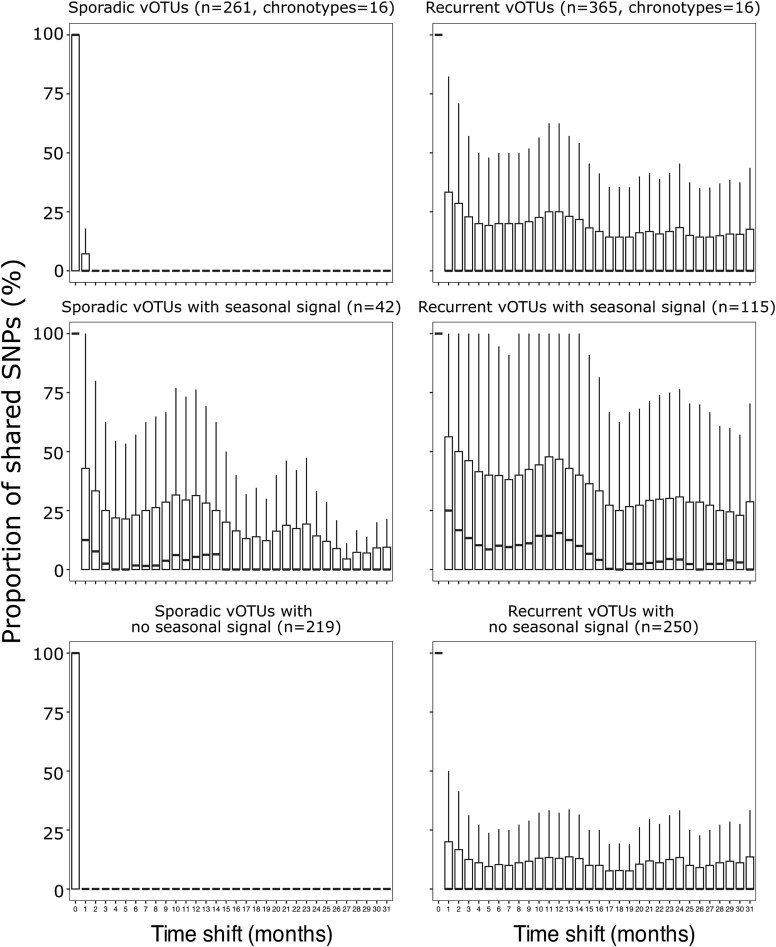
**Polymorphic profile comparisons in non-seasonal sporadic and seasonal annual recurrent vOTUs.** Sixteen non-seasonal (left panels) and 16 seasonal chronotypes (right panels) were selected, and the polymorphic profiles of their member vOTUs were compared pairwise between all potential combinations of the 27 available samples. The mean percentage of each unique monthly comparison (from 0 to 32 months) of shared SNPs for each vOTU was estimated. The resulting distribution of means was plotted as whisker plots (top panels). Using the Fisher G-test for time series, we determined which vOTUs have a distribution of shared means with a seasonal signal (mid panels). The vOTUs without a significant seasonal signal (bottom) are plotted for comparison purposes. In all the panels, outliers of the distributions were omitted to highlight the differences in the majority of the data.

Even though variance density correlates positively with coverage and RPKM (Fig. S8), when considering high-quality vOTUs with a minimum coverage of 10× across 90% of its length in at least one sample, we recovered variation profiles from most of the vOTUs comprising the total of our curated dataset (2997 out of 3090; ~97%). From these, we selected the vOTU members (n = 365) of 16 recurrent chronotypes, defined as those seasonal chronotypes that exhibited similar oscillatory dynamics and peaking abundances the same month for the years sampled, either annually peaking on summer or winter. As expected, the variant density of these vOTUs correlated significantly with their RPKM and coverage (Figs. S9, S10, S11), but it allowed us to explore whether genetically similar subpopulations may reoccur in a similar trend as their chronotype seasonality. By estimating the mean of the percentage of shared polymorphisms of a specific vOTU in all the potential time difference comparisons (0 to 32 months), we tested which vOTU polymorphic profiles displayed a seasonal signal (Figs. 7, S12, S13). We determined that the subpopulation genetic profiles of 31.5% vOTUs of the seasonal chronotypes showed a significant partial recurrence of genetic profiles that peaked in the 12 months pairwise comparisons. However, this signal fades out in profile comparisons of 16 months or more. We compared this pattern to vOTUs belonging to sporadic chronotypes, defined as those non-seasonal chronotypes presenting a sharp peak of abundance at a particular month, without any recurring pattern of similar abundances throughout the time series. We selected the vOTU members (n = 261) of 16 sporadic chronotypes and performed the same analysis. Only 16% of these vOTUs showed a seasonal signal, and 12-month recurrence median was less than half of that in the recurrent seasonal profiles. This low recurrence of genotype profiles of a few sporadic vOTUs might represent a residual or background signal which might not be filtered in the Fisher G-test of seasonality. These results support that a large fraction of vOTUs is subjected to a constant turnover of genetic population variants consistent with Red Queen Dynamics. However, a smaller fraction of vOTUs display patterns in which profiles of polymorphic populations are similar when time-periods of 12 months are compared.

## Discussion

In this study, we investigated the genomic commonalities and differences of viral populations that covaried through time at the WEC and the environmental factors shaping them. Our sampling strategy focused on the “free” viral size fraction (< 0.2 μm) at the surface (0–5 m). Overall, the viral community composition showed a remarkably stable annual periodicity in the WEC. A similar pattern was first reported at the SPOT [29], and later observed in Osaka Bay [31] where it was described that this annual trend was significantly correlated with the prokaryotic community composition and the seasonal environmental variables. A cycle of high-rate compositional changes from winter to summer and steadier transitions of viromes from summer to winter was described at WEC. As this cycle did not follow the same pace as the environmental changes, we hypothesize these oscillations are mostly driven by changes in host composition and productivity. This annual pattern may be more variable if shorter timescales, not captured by our monthly sampling, were analysed. Daily sampling of the T4-like myovirus major capsid protein gene in California coastal waters showed a viral community change rate of 0.4% per day [83]. A similar study at SPOT revealed high daily variability, especially during bloom decline [84]. This supports a differential rate of change in the surface viral community annual cycle. In contrast, 80 and 200 m diel virome sampling at the Bermuda Atlantic Time series suggested viral turnover occurs on timescales longer than 3 days [85]. The consistency of the annual oscillation in the virome rate of change and its differential pace at WEC suggests it is unlikely to be a coincidence driven by the monthly resolution of the time series. However, further analysis across other time series and higher-resolution sampling scales at WEC is needed to evaluate the broader prevalence of this signal.

To conduct a detailed analysis of the temporal patterns followed by vOTUs, we developed an unsupervised machine learning clustering method to classify the viral populations into chronotypes based on the comparisons of the distances between their abundance time series. This agnostic approach overcomes the major challenge of determining a meaningful threshold for partitioning the covariances by integrating an iterative search of an optimal number of clusters (*k*). Unsupervised machine learning distance-based clustering of time series provides a powerful method for classifying high-throughput datasets without prior knowledge of the classes [86]. The chronotype framework has proven successful in delineating the diversity of co-occurring populations, identifying genes involved in seasonal dynamics, and assessing the influence of environmental conditions on the assembly of these populations. Chronotypes did not exhibit specific taxonomic enrichments, possibly indicating a linear relationship with the diverse number of hosts that follow similar seasonal patterns of abundance. Further analysis, integrating host-derived data (such as amplicon or cellular fraction metagenome-assembled contigs) with viral chronotypes, or an improvement in accuracy of host-prediction from phage genomes [87], may offer better insights into these seasonal relationships and mechanisms of interaction.

AMGs play a crucial role in expanding host metabolic capacity and enhancing virus production in marine ecosystems [88,89]. These genes are fundamental genomic signatures across various locations and depths [90–94]. Here, we demonstrate that 2-OG(FeII) oxygenases and ferrochelatases, potential categories of AMGs, are enriched in seasonal chronotypes. 2-OG(FeII) oxygenases and ferrochelatases often co-occur in pelagiphages with podovirus morphology [68]. Phylogenetic analysis suggests that most of these predicted proteins have an origin in vOTUs taxonomically related to pelagiphages. SAR11 ecotypes show consistent and abundant seasonal patterns in 16S rRNA amplicon sequence variants in the WEC [66], which might explain an important fraction of the viral gene enrichments found in this study. The presence of these genes across seasonal chronotypes, with different patterns of correlation with environmental parameters, suggests a conserved enrichment in pelagiphages and taxonomically related vOTUs regardless of the season at which the specific host peaks. 2-OG(FeII) oxygenases catalyse diverse biochemical reactions, contributing to metabolite diversity and participate in sulfur and phosphate metabolism in microbial systems [82,95–97]. Because of their vast phylogenetic diversity, the specific functions and their role in seasonal virus-host interactions remain to be determined. Contrary to expectations, cyanophage AMGs, including photosystem II genes, were not enriched in seasonal chronotypes. This may be due to the dominance of pelagiphage-related sequences in co-occurring clusters or a relatively constant presence of these genes across both seasonal and non-seasonal chronotypes.

It has been previously shown that ocean viral populations showed a rapid and constant turnover of their genetic variant profiles to overcome the mutual selective pressure that viruses and their hosts exert on each other, suggestive of Red Queen dynamics [29]. Here, we analysed whether this pattern was consistent in the WEC. Our results showed that most of the viral populations followed a similar trend, in which a specific polymorphism profile increases to a maximum and then decays within a few months. In contrast to the trend reported at SPOT [29], in which the median of the shared polymorphism distribution decays down to almost 0% before and after 12 months, in the WEC the medians stabilize at ~50% before and after five months. This pattern is the integral of dynamics of individual profiles which display variable patterns of rise and decay. When we subset and analyse the most consistent seasonal chronotypes (annual recurrent with similar abundances than previous years), we found that some populations have a strong recurrent signal of a shared collection of polymorphisms when profiles 12-months apart are compared. This signal fades out as comparisons are done for longer periods of time. These results suggest that in the WEC, some phage populations might have a slower rate of change, specifically in an annual scale instead of monthly, although still consistent with Red Queen dynamics as these similarities faded out as the years progressed.

This study presents an innovative framework to interrogate the temporal patterns of viruses in the surface ocean. Our findings reveal that the stable seasonality observed in the viral community can be deconstructed into seasonal and a smaller fraction of non-seasonal chronotypes. We suggest that the seasonal patterns of viral chronotypes are adapted to couple their productivity with that of their recurrent seasonal hosts, such as SAR11, while non-seasonal chronotypes experience an increase in abundance triggered by specific conditions, leading to short-term negative density-dependent selection, likely involving copiotrophic hosts. Both mechanisms broadly follow the Kill-the-Winner model [98], but operate at different time scales. The significant association of 2-OG(FeII) oxygenases and ferrochelatases with seasonal chronotypes suggests that a diverse array of versatile AMG functions within these genes have been evolutionarily selected as an adaptive strategy to sustain the replication and production of viruses at a seasonal scale. The robustness of the time series clustering method introduced in this study will facilitate the generation of highly defined co-occurring longitudinal units in other molecular datasets. As demonstrated by this study, such an approach will contribute to uncovering ecological and evolutionary patterns in longitudinal data.

## Supplementary Material

WEC_Viromes_resubm_V7_Sup_FinalSubm_wrae216

## Data Availability

Short read viral metagenomes are deposited in NCBI under the Bioproject PRJNA804019.

## References

[1] Suttle CA . Viruses in the sea. *Nature* 2005;437:356–61. 10.1038/nature04160 16163346

[2] Kristensen DM , Mushegian AR, Dolja VV et al. New dimensions of the virus world discovered through metagenomics. *Trends Microbiol* 2010;18:11–9. 10.1016/j.tim.2009.11.003 19942437 PMC3293453

[3] Falkowski PG , Fenchel T, Delong EF. The microbial engines that drive Earth's biogeochemical cycles. *Science* 2008;320:1034–9. 10.1126/science.1153213 18497287

[4] Fenchel T . Marine plankton food chains. *Annu Rev Ecol Evol Syst* 1988;19:19–38. 10.1146/annurev.es.19.110188.000315

[5] Carpenter LJ , Nightingale PD. Chemistry and release of gases from the surface ocean. *Chem Rev* 2015;115:4015–34. 10.1021/cr5007123 25811324

[6] Carlson CA , Ducklow HW, Michaels AF. Annual flux of dissolved organic carbon from the euphotic zone in the northwestern Sargasso Sea. *Nature* 1994;371:405–8. 10.1038/371405a0

[7] Carlson CA , Hansell DA, Nelson NB et al. Dissolved organic carbon export and subsequent remineralization in the mesopelagic and bathypelagic realms of the North Atlantic basin. *Deep Sea Res Part II Top Stud Oceanogr* 2010;57:1433–45. 10.1016/j.dsr2.2010.02.013

[8] Wilhelm SW , Suttle CA. Viruses and nutrient cycles in the sea: viruses play critical roles in the structure and function of aquatic food webs. *Biosci* 1999;49:781–8.

[9] Weinbauer MG . Ecology of prokaryotic viruses. *FEMS Microbiol Rev* 2004;28:127–81. 10.1016/j.femsre.2003.08.001 15109783

[10] Fuhrman JA . Marine viruses and their biogeochemical and ecological effects. *Nature* 1999;399:541–8. 10.1038/21119 10376593

[11] Breitbart M . Marine viruses: truth or dare. *Annu Rev Mar Sci* 2012;4:425–48. 10.1146/annurev-marine-120709-142805 22457982

[12] Forterre P . The virocell concept and environmental microbiology. *ISME J* 2013;7:233–6. 10.1038/ismej.2012.110 23038175 PMC3554396

[13] Tran PQ , Anantharaman K. Biogeochemistry goes viral: towards a multifaceted approach to study viruses and biogeochemical cycling. *mSystems* 2021;6:e01138–21. 10.1128/msystems.01138-21 34636672 PMC8510517

[14] Brum JR , Hurwitz BL, Schofield O et al. Seasonal time bombs: dominant temperate viruses affect Southern Ocean microbial dynamics. *ISME J* 2016;10:437–49. 10.1038/ismej.2015.125 26296067 PMC4737935

[15] Diaz BP , Knowles B, Johns CT et al. Seasonal mixed layer depth shapes phytoplankton physiology, viral production, and accumulation in the North Atlantic. *Nat Commun* 2021;12:6634. 10.1038/s41467-021-26836-1 34789722 PMC8599477

[16] Chow C-ET , Fuhrman JA. Seasonality and monthly dynamics of marine myovirus communities. *Environ Microbiol* 2012;14:2171–83. 10.1111/j.1462-2920.2012.02744.x 22507260

[17] Roux S , Chan LK, Egan R et al. Ecogenomics of virophages and their giant virus hosts assessed through time series metagenomics. *Nat Commun* 2017;8:858. 10.1038/s41467-017-01086-2 29021524 PMC5636890

[18] Ahlgren NA , Perelman JN, Yeh YC et al. Multi-year dynamics of fine-scale marine cyanobacterial populations are more strongly explained by phage interactions than abiotic, bottom-up factors. *Environ Microbiol* 2019;21:2948–63. 10.1111/1462-2920.14687 31106939

[19] Danovaro R , Corinaldesi C, Dell'Anno A et al. Marine viruses and global climate change. *FEMS Microbiol Rev* 2011;35:993–1034. 10.1111/j.1574-6976.2010.00258.x 21204862

[20] Breitbart M , Salamon P, Andresen B et al. Genomic analysis of uncultured marine viral communities. *Proc Natl Acad Sci USA* 2002;99:14250–5. 10.1073/pnas.202488399 12384570 PMC137870

[21] Brum JR , Ignacio-Espinoza JC, Roux S et al. Patterns and ecological drivers of ocean viral communities. *Science* 2015;348:1261498. 10.1126/science.1261498 25999515

[22] Roux S , Brum JR, Dutilh BE et al. Ecogenomics and potential biogeochemical impacts of globally abundant ocean viruses. *Nature* 2016;537:689–93. 10.1038/nature19366 27654921

[23] Zayed AA , Wainaina JM, Dominguez-Huerta G et al. Cryptic and abundant marine viruses at the evolutionary origins of Earth’s RNA virome. *Science* 2022;376:156–62. 10.1126/science.abm5847 35389782 PMC10990476

[24] Gregory AC , Zayed AA, Conceição-Neto N et al. Marine DNA viral macro-and microdiversity from pole to pole. *Cell* 2019;177:1109–23. 10.1016/j.cell.2019.03.040 31031001 PMC6525058

[25] Stern A , Sorek R. The phage-host arms race: shaping the evolution of microbes. *BioEssays* 2011;33:43–51. 10.1002/bies.201000071 20979102 PMC3274958

[26] Sieradzki ET , Ignacio-Espinoza JC, Needham DM et al. Dynamic marine viral infections and major contribution to photosynthetic processes shown by spatiotemporal picoplankton metatranscriptomes. *Nat Commun* 2019;10:10. 10.1038/s41467-019-09106-z 30862830 PMC6414667

[27] Aylward FO , Boeuf D, Mende DR et al. Diel cycling and long-term persistence of viruses in the ocean's euphotic zone. *Proc Natl Acad Sci USA* 2017;114:11446–51. 10.1073/pnas.1714821114 29073070 PMC5663388

[28] Hevroni G , Flores-Uribe J, Beja O et al. Seasonal and diel patterns of abundance and activity of viruses in the Red Sea. *Proc Natl Acad Sci USA* 2020;117:29738–47. 10.1073/pnas.2010783117 33172994 PMC7703586

[29] Ignacio-Espinoza JC , Ahlgren NA, Fuhrman JA. Long-term stability and red queen-like strain dynamics in marine viruses. *Nature Microbiol* 2020;5:265–71. 10.1038/s41564-019-0628-x 31819214

[30] Luo E , Eppley JM, Romano AE et al. Double-stranded DNA virioplankton dynamics and reproductive strategies in the oligotrophic open ocean water column. *ISME J* 2020;14:1304–15. 32060418 10.1038/s41396-020-0604-8 PMC7174320

[31] Tominaga K , Ogawa-Haruki N, Nishimura Y et al. Prevalence of viral frequency-dependent infection in coastal marine prokaryotes revealed using monthly time series virome analysis. *mSystems* 2023;8:e00931–22. 10.1128/msystems.00931-22 36722950 PMC9948707

[32] Parsons RJ , Breitbart M, Lomas MW et al. Ocean time series reveals recurring seasonal patterns of virioplankton dynamics in the northwestern sargasso sea. *ISME J* 2012;6:273–84. 10.1038/ismej.2011.101 21833038 PMC3260494

[33] Pagarete A , Chow CE, Johannessen T et al. Strong seasonality and interannual recurrence in marine myovirus communities. *Appl Environ Microbiol* 2013;79:6253–9. 10.1128/AEM.01075-13 23913432 PMC3811205

[34] Brockhurst MA , Chapman T, King KC et al. Running with the red queen: the role of biotic conflicts in evolution. *Proc R Soc B* 2014;281:20141382. 10.1098/rspb.2014.1382 PMC4240979 25355473

[35] Dart E , Fuhrman JA, Ahlgren NA. Diverse marine T4-like cyanophage communities are primarily comprised of low-abundance species including species with distinct seasonal, persistent, occasional, or sporadic dynamics. *Viruses* 2023;15:581. 10.3390/v15020581 36851794 PMC9960396

[36] Smyth TJ , Fishwick JR, Al-Moosawi L et al. A broad spatio-temporal view of the Western English Channel observatory. *J Plankton Res* 2010;32:585–601. 10.1093/plankt/fbp128

[37] Gilbert JA , Field D, Swift P et al. The seasonal structure of microbial communities in the Western English Channel. *Environ Microbiol* 2009;11:3132–9. 10.1111/j.1462-2920.2009.02017.x 19659500

[38] Caporaso JG , Paszkiewicz K, Field D et al. The Western English Channel contains a persistent microbial seed bank. *ISME J* 2012;6:1089–93. 10.1038/ismej.2011.162 22071345 PMC3358019

[39] Gilbert JA , Steele JA, Caporaso JG et al. Defining seasonal marine microbial community dynamics. *ISME J* 2012;6:298–308. 10.1038/ismej.2011.107 21850055 PMC3260500

[40] Tarran GA , Bruun JT. Nanoplankton and picoplankton in the Western English Channel: abundance and seasonality from 2007-2013. *Prog Oceanogr* 2015;137:446–55. 10.1016/j.pocean.2015.04.024

[41] McEvoy AJ , Atkinson A, Airs RL et al. The Western Channel observatory: a century of oceanographic, chemical and biological data compiled from pelagic and benthic habitats in the Western English Channel. *Earth Syst Sci Data Discuss* 2023;2023:1–42.

[42] McEvoy A. , Atkinson A. The Western Channel observatory: a century of oceanographic, chemical and biological data compiled from pelagic and benthic habitats in the Western English Channel 1903 - 2022. Biomed Instrum Technol 2023;57:163–70. 10.2345/0899-8205-57.4.163

[43] Wilkinson B , Jones O, Rees A et al. Fluorometric chlorophyll-a measurements from CTD niskin collected from depth profiles at station L4 in the Western Channel observatory (WCO) from 1992 up until end of 2022. *NERC EDS British Oceanographic Data Centre NOC* 2023. 10.5285/0a1cac9c-1217-247e-e063-6c86abc0507c

[44] Warwick-Dugdale J , Solonenko N, Moore K et al. Long-read viral metagenomics captures abundant and microdiverse viral populations and their niche-defining genomic islands. *PeerJ* 2019;7:e6800. 10.7717/peerj.6800 31086738 PMC6487183

[45] John SG , Mendez CB, Deng L et al. A simple and efficient method for concentration of ocean viruses by chemical flocculation. *Environ Microbiol Rep* 2011;3:195–202. 10.1111/j.1758-2229.2010.00208.x 21572525 PMC3087117

[46] Deng L , Ignacio-Espinoza JC, Gregory AC et al. Viral tagging reveals discrete populations in *Synechococcus* viral genome sequence space. *Nature* 2014;513:242–5. 10.1038/nature13459 25043051

[47] Bushnell B , Madsen MT, O'cdorisio T et al. BBTools software package. 2014;4:38. 10.1186/s13550-014-0038-2. http://bbtools.jgi.doe.gov.

[48] Nurk S , Meleshko D, Korobeynikov A et al. metaSPAdes: a new versatile metagenomic assembler. *Genome Res* 2017;27:824–34. 10.1101/gr.213959.116 28298430 PMC5411777

[49] Roux S , Enault F, Hurwitz BL et al. VirSorter: mining viral signal from microbial genomic data. *PeerJ* 2015;3:e985. 10.7717/peerj.985 26038737 PMC4451026

[50] Nayfach S , Camargo AP, Schulz F et al. CheckV assesses the quality and completeness of metagenome-assembled viral genomes. *Nat Biotecnol* 2021;39:578–85. 10.1038/s41587-020-00774-7 PMC8116208 33349699

[51] Cook R , Brown N, Redgwell T et al. INfrastructure for a PHAge REference database: identification of large-scale biases in the current collection of cultured phage genomes. *Phage* 2021;2:214–23. 10.1089/phage.2021.0007 36159887 PMC9041510

[52] Bin Jang H , Bolduc B, Zablocki O et al. Taxonomic assignment of uncultivated prokaryotic virus genomes is enabled by gene-sharing networks. *Nat Biotechnol* 2019;37:632–9. 10.1038/s41587-019-0100-8 31061483

[53] Bouras G , Nepal R, Houtak G et al. Pharokka: a fast scalable bacteriophage annotation tool. *BMC Bioinf* 2023;39:776. 10.1093/bioinformatics/btac776 PMC9805569 36453861

[54] Shaffer M , Borton MA, McGivern BB et al. DRAM for distilling microbial metabolism to automate the curation of microbiome function. *Nucleic Acids Res* 2020;48:8883–900. 10.1093/nar/gkaa621 32766782 PMC7498326

[55] Langmead B , Salzberg SL. Fast gapped-read alignment with bowtie 2. *Nat Methods* 2012;9:357–9. 10.1038/nmeth.1923 22388286 PMC3322381

[56] Aroney STN , Newell RJP, Nissen J et al. CoverM: Read coverage calculator for metagenomics. 2024 (Version 0.7.0) [Computer software]. 10.5281/zenodo.10531253

[57] Mölder F , Jablonski KP, Letcher B et al. Sustainable data analysis with Snakemake. *F1000Research* 2021;10:10. 10.12688/f1000research.29032.1 PMC8114187 34035898

[58] Auladell A , Sánchez P, Sánchez O et al. Long-term seasonal and interannual variability of marine aerobic anoxygenic photoheterotrophic bacteria. *ISME J* 2019;13:1975–87. 10.1038/s41396-019-0401-4 30914777 PMC6776013

[59] Ahdesmaki M, Fokianos K, Strimmer K. GeneCycle: Identification of Periodically Expressed Genes. R package version 1.1.5, 2021. https://CRAN.R-project.org/package=GeneCycle

[60] Montero P , Vilar JA. TSclust: an R package for time series clustering. *J Stat Softw* 2015;62:1–43.

[61] Zeileis A , Grothendieck G. Zoo: S3 infrastructure for regular and irregular time series. J Stat Softw 2005;14:1–27.

[62] Shade A , Gregory Caporaso J, Handelsman J et al. A meta-analysis of changes in bacterial and archaeal communities with time. *ISME J* 2013;7:1493–506. 10.1038/ismej.2013.54 23575374 PMC3721121

[63] Fuhrman JA , Cram JA, Needham DM. Marine microbial community dynamics and their ecological interpretation. *Nat Rev Microbiol* 2015;13:133–46. 10.1038/nrmicro3417 25659323

[64] R Core Team. R: a language and environment for statistical computing. Vienna, Austria ; https://www.R-project.org/.

[65] Roesch A , Schmidbauer H, Roesch MA. Package ‘WaveletComp.’ 2014.

[66] Bolaños LM , Tait K, Somerfield PJ et al. Influence of short and long term processes on SAR11 communities in open ocean and coastal systems. *ISME Commun* 2022;2:116. 10.1038/s43705-022-00198-1 37938786 PMC9723719

[67] Revelle W , Revelle MW. Package ‘psych’. CRAN 2015;337:161–165.

[68] Wittmers F , Needham DM, Hehenberger E et al. Genomes from uncultivated pelagiphages reveal multiple phylogenetic clades exhibiting extensive auxiliary metabolic genes and cross-family multigene transfers. *mSystems* 2022;7:e01522–1. 10.1128/msystems.01522-21 35972150 PMC9599517

[69] Zhao Y , Temperton B, Thrash JC et al. Abundant SAR11 viruses in the ocean. *Nature* 2013;494:357–60. 10.1038/nature11921 23407494

[70] Zhao Y , Qin F, Zhang R et al. Pelagiphages in the Podoviridae family integrate into host genomes. *Environ Microbiol* 2019;21:1989–2001. 10.1111/1462-2920.14487 30474915

[71] Buchholz HH , Michelsen ML, Bolaños LM et al. Efficient dilution-to-extinction isolation of novel virus-host model systems for fastidious heterotrophic bacteria. *ISME J* 2021;15:1585–98. 10.1038/s41396-020-00872-z 33495565 PMC8163748

[72] Buchholz HH , Michelsen M, Parsons RJ et al. Draft genome sequences of Pelagimyophage Mosig EXVC030M and Pelagipodophage Lederberg EXVC029P, isolated from Devil’s hole, Bermuda. *Genome announcements* 2021;10:10–128. 10.1128/MRA.01325-20 PMC7892664 33602731

[73] Buchholz HH , Bolaños LM, Bell AG et al. Novel pelagiphage isolate *Polarivirus skadi* is a polar specialist that dominates SAR11-associated bacteriophage communities at high latitudes. *ISME J* 2023;17:1660–70. 10.1038/s41396-023-01466-1 37452097 PMC10504331

[74] Notredame C , Higgins DG, Heringa J. T-coffee: a novel method for fast and accurate multiple sequence alignment. *J Mol Biol* 2000;302:205–17. 10.1006/jmbi.2000.4042 10964570

[75] Minh BQ , Schmidt HA, Chernomor O et al. IQ-TREE 2: new models and efficient methods for phylogenetic inference in the genomic era. *Mol Biol Evol* 2020;37:1530–4. 10.1093/molbev/msaa015 32011700 PMC7182206

[76] Paradis E , Schliep K. Ape 5.0: an environment for modern phylogenetics and evolutionary analyses in R. *Bioinformatics* 2019;35:526–8. 10.1093/bioinformatics/bty633 30016406

[77] Yu G , Smith DK, Zhu H et al. Ggtree: an R package for visualization and annotation of phylogenetic trees with their covariates and other associated data. *Methods Ecol Evol* 2017;8:28–36. 10.1111/2041-210X.12628

[78] Li H , Handsaker B, Wysoker A et al. The sequence alignment/map format and SAMtools. *Bioinformatics* 2009;25:2078–9. 10.1093/bioinformatics/btp352 19505943 PMC2723002

[79] Danecek P , Bonfield JK, Liddle J et al. Twelve years of SAMtools and BCFtools. Gigascience 2021:10(2), giab008, 10.1093/gigascience/giab008. 33590861 PMC7931819

[80] Wickham H . ggplot2. *WIREs Computational Stats* 2011;3:180–5. 10.1002/wics.147

[81] Millard A , Clokie MR, Shub DA et al. Genetic organization of the psbAD region in phages infecting marine *Synechococcus* strains. *Proc Natl Acad Sci USA* 2004;101:11007–12. 10.1073/pnas.0401478101 15263091 PMC503734

[82] Jia B , Jia X, Kim KH et al. Integrative view of 2-oxoglutarate/Fe (II)-dependent oxygenase diversity and functions in bacteria. *Biochim Biophys Acta Gen Subj* 2017;1861:323–34. 10.1016/j.bbagen.2016.12.001 27919802

[83] Needham DM , Chow C-ET, Cram JA et al. Short-term observations of marine bacterial and viral communities: patterns, connections and resilience. *ISME J* 2013;7:1274–85. 10.1038/ismej.2013.19 23446831 PMC3695287

[84] Needham DM , Sachdeva R, Fuhrman JA. Ecological dynamics and co-occurrence among marine phytoplankton, bacteria and myoviruses shows microdiversity matters. *ISME J* 2017;11:1614–29. 10.1038/ismej.2017.29 28398348 PMC5520149

[85] Warwick-Dugdale J , Tian F, Michelsen ML et al. Long-read powered viral metagenomics in the oligotrophic Sargasso Sea. *Nat Commun* 2024;15:4089. 10.1038/s41467-024-48300-6 38744831 PMC11094077

[86] Aghabozorgi S , Shirkhorshidi AS, Wah TY. Time series clustering-a decade review. *Inf Syst* 2015;53:16–38. 10.1016/j.is.2015.04.007

[87] Iuchi H , Kawasaki J, Kubo K et al. Bioinformatics approaches for unveiling virus-host interactions. *Comput Struct Biotechnol J* 2023;21:1774–84. 10.1016/j.csbj.2023.02.044 36874163 PMC9969756

[88] Breitbart MY , Thompson LR, Suttle CA et al. Exploring the vast diversity of marine viruses. *Oceanography* 2007;20:135–9. 10.5670/oceanog.2007.58

[89] Breitbart M . Marine viruses: truth or dare. *Annu Rev Mar Sci* 2012;4:425–48. 10.1146/annurev-marine-120709-142805 22457982

[90] Hurwitz BL , Brum JR, Sullivan MB. Depth-stratified functional and taxonomic niche specialization in the ‘core’ and ‘flexible’Pacific Ocean Virome. *ISME J* 2015;9:472–84. 10.1038/ismej.2014.143 25093636 PMC4303639

[91] Hurwitz BL , U’Ren JM. Viral metabolic reprogramming in marine ecosystems. *Curr Opin Microbiol* 2016;31:161–8. 10.1016/j.mib.2016.04.002 27088500

[92] Coutinho FH , Silveira CB, Gregoracci GB et al. Marine viruses discovered via metagenomics shed light on viral strategies throughout the oceans. *Nat Commun* 2017;8:15955. 10.1038/ncomms15955 28677677 PMC5504273

[93] Coutinho FH , Silveira CB, Sebastián M et al. Water mass age structures the auxiliary metabolic gene content of free-living and particle-atached deep ocean viral communities. *Microbiome* 2023;11:1–4. 10.1186/s40168-023-01547-5 37237317 PMC10224230

[94] Luo X-Q , Wang P, Li JL et al. Viral community-wide auxiliary metabolic genes differ by lifestyles, habitats, and hosts. *Microbiome* 2022;10:190. 10.1186/s40168-022-01384-y 36333738 PMC9636769

[95] Herr CQ , Hausinger RP. Amazing diversity in biochemical roles of Fe (II)/2-oxoglutarate oxygenases. *Trends Biochem Sci* 2018;43:517–32. 10.1016/j.tibs.2018.04.002 29709390 PMC6014900

[96] Islam MS , Leissing TM, Chowdhury R et al. 2-Oxoglutarate-dependent oxygenases. *Annu Rev Biochem* 2018;87:585–620. 10.1146/annurev-biochem-061516-044724 29494239

[97] Hausinger RP . Biochemical Diversity of 2-Oxoglutarate-Dependent Oxygenases. London: Royal Society of Chemistry. 2015, Biochemical Diversity of 2-Oxoglutarate-Dependent Oxygenases, 10.1039/9781782621959-00001

[98] Thingstad TF , Lignell R. Theoretical models for the control of bacterial growth rate, abundance, diversity and carbon demand. *Aquat Microb Ecol* 1997;13:19–27. 10.3354/ame013019

