# Supplementary material for: Metagenomic time series reveals a Western English Channel viral community dominated by members with strong seasonal signals
Source: ISME J. 2024 Oct 23;18(1):wrae216. doi: 10.1093/ismejo/wrae216 (PMC11561400; doi:10.1093/ismejo/wrae216)
Supplement: WEC_Viromes_resubm_V7_Sup_FinalSubm_wrae216 [file wec_viromes_resubm_v7_sup_finalsubm_wrae216.docx]

Metagenomic time-series reveals a Western English Channel viral community dominated by members with strong seasonal signals

Luis M. Bolaños^1*^, Michelle Michelsen^1^, Ben Temperton^1*^

1 School of Biosciences, University of Exeter, Exeter, UK

***Corresponding Author**

l.bolanos-avellaneda@exeter.ac.uk

b.temperton@exeter.ac.uk

Supplementary Material

Supplementary methods:

**Unsupervised machine learning framework to generate chronotypes: ChronoClustR.**

We present an unsupervised machine learning framework for generating chronotypes, named ChronoClustR. Time-series clustering is an essential framework across multiple disciplines and has undergone constant development (Aghabozorgi *et al.*, 2015). In the era of big data, unsupervised machine learning clustering methods have played a fundamental role in generating patterns and classes when no a priori knowledge is available. In this study, we propose an alternative method to generate highly defined clusters of time-series datasets (Fig. 1). Our method provides important benefits - the most important is the automatic determination of optimal clusters based on distances that allow us to capture local and global patterns. From the user perspective, multiple advantages exist such as: interpretability, scalability, flexibility in data types, and few pre-processing requirements. The following pipeline was developed in R v4.3.

**User input:**

This pipeline requires an equidistant set of time-series with no missing values. The user should input the following:

1. A data frame of the vOTU RPKM (or any other value) by timepoint (sample).
2. Metadata with the corresponding date.
3. Number of bootstrap iterations (this value should be greater than 1).
4. Size of the subsample (default = 100).
5. The starting date.
6. The end date.
7. Specify the frequency or number of timepoints.

**Processing of individual replicates:**

**Subsampling:** Randomly subsample (without replacement) the collection of time-series using the user-specified size. The user may consider estimating a subsample size that generates an exact integer of subsamples (to cover all time-series within the dataset) or the one that generates the smallest residue (Fig. 1, step 6).

**Unsupervised Clustering of Each Subsample** (Default Size 100): A function named "centroid_generator" clusters each subsample using the TSclust algorithm (Fig. 1, step 7) with the following default parameters:

1. Type (clustering method): Partitional
2. k (number of clusters to evaluate): 3 to (n-1)
3. Distance: Euclidean
4. Centroid: Partitioning around medoids (PAM)

Any of the above parameters can be adjusted to fulfil user requirements. The options are those used by the TSclust algorithm [60]. After generating the clustering of each subsample, the "centroid_generator" function will retrieve the best number of clusters based on the maximum Silhouette score for each tested number of clusters (*k*). This function will then recalculate the clusters for this *k* and retrieve a list of centroids and their associated cluster members.

**Unsupervised Clustering of Centroids and Optimized *k*:** The centroids generated from the clusters of all subsamples are extracted and concatenated. This collection of centroids is clustered using the k-means algorithm, testing all potential numbers of clusters (*k*) from 1 to n-1. The generated within-cluster sum of square for each computed cluster for each value of *k* is extracted (Fig. 1, step 8). Based on the curve generated from the relationship between the total within-cluster sum of squares and *k*, we define the optimal number of clusters by estimating the closest value of *k* to the first derivative (slope of the tangent line). This first derivative is calculated on the fitted curve estimated on the relationship between the number of clusters and its within-cluster sums of squares (Fig. 1, step 9).

**Unsupervised clustering with an optimized *k:*** Once the best *k* is estimated based on the centroids of the subsamples, this *k* is used to generate three types of clustering (k-means, hierarchical clustering, and TSclust) on the original dataset (Fig. 1, step 10).

**Bootstrap:** The above steps are coded to be repeated as many times as indicated by the user (Fig. 1, step 11). Warning: As the number of iterations increase, the memory needed to generate the co-occurrence matrix also increases, limiting the successful analysis by the memory capacity of the user’s computing infrastructure.

**Co-occurrence matrix and chronotype generation**: After *n* replicates of bootstrap, the configurations of the different clusters of each replicate are used to generate a co-occurrence matrix (Fig. 1, step 12). The user can choose to use any of the three clustering methods from step 10. The co-occurrence values are normalized to the number of iterations (*n*) used for the bootstrap. Finally, a dendrogram is generated using the hclust function, and the maximum optimal number of clusters from all the iterations is used to partition the dendrogram. Each partition will be considered a chronotype (Fig. 1, step 13).

R scripts and data used to generate chronotypes are available at https://github.com/lbolanos32/WEC_Chronotypes_2024.

**Bray-Curtis dissimilarity and environmental Euclidean distances**

To corroborate the medoid clustering patterns based on their correlations with the different environmental variables, a constrained ordination was generated. The Canonical Analysis of Principle Coordinates (CAP) of medoid temporal profiles was generated based on the Bray–Curtis dissimilarities estimated using the derived medoid rpkm values as described in [63]. To calculate the contribution of each environmental variable to the ordination variance. the squared correlations (R² values) of the biplot scores for each environmental variable was calculated. These were normalized to sum 1 and visualized as a barplot.

**Polymorphism profiles**

The decay model of the distribution median of shared polymorphisms through time was derived independently for the negative values (before the fixed profile) and the positive values (after the fixed profile). The mathematical formulation has the same structure for both sets of values:

$$median percentage = a \left( \frac{1}{1+\frac{1}{1+exp\left( -k\left( Month Difference + 5 \right) \right)}} \right) * exp (b*\left( Month Difference +5 \right))$$

*a* is a scaling factor = 0.5 to adjust the magnitude to half

*b* is the exponential growth term (rate of growth) = 0.1 value was set to have a relatively slow growth value.

*K* controls the steepness of the transition to the exponential phase = 0.5 to set a moderate rate of transition.

Month Difference = independent variable to estimate the median percentage.

5 in the formula determines the point of transition for the sigmoid function. This can be adjusted.

The R-squared value was calculated as follows: 1 - (RSS/ SS).

Where RSS is the sum of the squared residuals and SS is the sum of the squared deviations of the observed values from their mean.

**Supplementary Figures and Tables:**

**Table S1: Environmental metadata and virome accession (raw short reads).**

| **Sample** | **Date** | **Date_wav** | **SRA (NCBI)** | **Temp (°C)** | **Fluor (V)** | **Dens (kg.m3)** | **Sal (PSU)** | **O2 (uM)** |
| --- | --- | --- | --- | --- | --- | --- | --- | --- |
| 2018-Nov | 20/11/2018 | 07/11/2018 | SRR18164412 | 13.3915 | 0.7567 | 1026.437 | 35.1443 | 245.262 |
| 2018-Dec | 04/12/2018 | 07/12/2018 | SRR18164411 | 12.1169 | 0.7576 | 1026.2895 | 34.6282 | 253.638 |
| 2019-Jan | 14/01/2019 | 07/01/2019 | SRR18164400 | 11.011 | 0.6565 | 1026.7145 | 34.9094 | 260.321 |
| 2019-Feb | 11/02/2019 | 07/02/2019 | SRR18164392 | 9.3312 | 0.434 | 1026.4479 | 34.1984 | 270.148 |
| 2019-Mar | 11/03/2019 | 07/03/2019 | SRR18164391 | 10.0049 | 0.4015 | 1026.9742 | 35.0154 | 267.477 |
| 2019-Apr | 01/04/2019 | 07/04/2019 | SRR18164390 | 10.3946 | 0.4577 | 1026.947 | 35.067 | 270.54 |
| 2019-May | 07/05/2019 | 07/05/2019 | SRR18164389 | 11.4882 | 0.3845 | 1026.701 | 35.0051 | 289.791 |
| 2019-Jun | 03/06/2019 | 07/06/2019 | SRR18164388 | 13.6776 | 0.2984 | 1026.3111 | 35.0588 | 278.34 |
| 2019-Jul | 22/07/2019 | 07/07/2019 | SRR18164387 | 17.33 | 0.5402 | 1025.6144 | 35.2189 | 224.859 |
| 2019-Aug | 12/08/2019 | 07/08/2019 | SRR18164386 | 16.9253 | 0.4441 | 1025.7414 | 35.2582 | 242.563 |
| 2019-Sep | 02/09/2019 | 07/09/2019 | SRR18164410 | 16.3641 | 0.5957 | 1025.8784 | 35.2647 | 239.814 |
| 2019-Oct | 02/10/2019 | 07/10/2019 | - | 16.1277 | 0.6609 | 1025.9259 | 35.2569 | 229.279 |
| 2019-Nov | 05/11/2019 | 07/11/2019 | SRR18164409 | 13.7882 | 0.6881 | 1025.7409 | 34.3507 | 228.511 |
| 2019-Dec | 02/12/2019 | 07/12/2019 | SRR18164408 | 12.0838 | 0.4746 | 1026.4812 | 34.8668 | 237.124 |
| 2020-Jan | 20/01/2020 | 07/01/2020 | SRR18164407 | 9.8135 | - | 1026.43 | 34.2763 | 239.545 |
| 2020-Feb | 05/02/2020 | 07/02/2020 | SRR18164406 | 9.7321 | - | 1026.5673 | 34.4347 | 240.232 |
| 2020-Mar | 02/03/2020 | 07/03/2020 | SRR18164405 | 9.1438 | - | 1026.0072 | 33.5967 | 265.805 |
| 2020-Apr | 07/04/2020 | 07/04/2020 | - | 9.9763 | - | 1026.9247 | 34.9476 | 283.251 |
| 2020-May | 04/05/2020 | 07/05/2020 | - | 11.1393 | 0.7241 | 1026.7412 | 34.9736 | 276.215 |
| 2020-Jun | 08/06/2020 | 07/06/2020 | - | 12.9412 | 0.9564 | 1026.4234 | 35.0108 | 256.979 |
| 2020-Jul | 07/07/2020 | 07/07/2020 | SRR18164404 | 13.731 | 3.657 | 1026.2237 | 34.9597 | 271.245 |
| 2020-Aug | 10/08/2020 | 07/08/2020 | SRR18164403 | 16.5421 | 1.6689 | 1025.6532 | 35.0254 | 255.713 |
| 2020-Sep | 07/09/2020 | 07/09/2020 | SRR18164402 | 15.9492 | 0.9631 | 1025.7824 | 35.0157 | 235.425 |
| 2020-Oct | 12/10/2020 | 07/10/2020 | SRR18164401 | 14.7043 | 1.6795 | 1025.8094 | 34.6917 | 201.806 |
| 2020-Nov | 09/11/2020 | 07/11/2020 | SRR18164399 | 13.6133 | 1.5318 | 1026.2712 | 34.9894 | 214.929 |
| 2020-Dec | 07/12/2020 | 07/12/2020 | SRR18164398 | 11.9147 | 0.6191 | 1026.5668 | 34.9362 | 220.044 |
| 2021-Jan | 11/01/2021 | 07/01/2021 | SRR18164397 | 9.5617 | 1.0781 | 1026.7136 | 34.5838 | 236.779 |
| 2021-Feb | 08/02/2021 | 07/02/2021 | - | 9.0447 | 0.7727 | 1026.5613 | 34.2846 | 234.979 |
| 2021-Mar | 08/03/2021 | 07/03/2021 | SRR18164396 | 8.4951 | 0.7257 | 1026.8666 | 34.5622 | 226.851 |
| 2021-Apr | 13/04/2021 | 07/04/2021 | SRR18164395 | 9.334 | 1.0157 | 1027.0553 | 34.9701 | 248.859 |
| 2021-May | 12/05/2021 | 07/05/2021 | SRR18164394 | 10.8083 | 0.9175 | 1026.8078 | 34.983 | 259.301 |
| 2021-Jun | 07/06/2021 | 07/06/2021 | SRR18164393 | 14.2321 | 1.6498 | 1025.5136 | 34.1774 | 233.462 |

**Table S1 (continuation)**

| **Sample** | **NO_2_ (uM)** | **NO_3_+NO_2_ (uM)** | **NH_4_ (uM)** | **SiO_4_ (uM)** | **PO_4_ (uM)** | **Chl *a* (ugL^-1^)** |
| --- | --- | --- | --- | --- | --- | --- |
| 2018-Nov | 0.15 | 3.51 | 0.19 | 2.66 | 0.4 | 0.75 |
| 2018-Dec | 0.26 | 7.5 | 0.35 | 4.66 | 0.45 | 0.61 |
| 2019-Jan | 0.13 | 7.53 | 0.19 | 4.02 | 0.5 | 0.29 |
| 2019-Feb | 0.3 | 11.8 | 0.3 | 4.82 | 0.57 | 0.47 |
| 2019-Mar | 0.36 | 6.88 | 0.1 | 2.67 | 0.52 | 0.43 |
| 2019-Apr | 0.28 | 5.75 | 0.26 | 1.9 | 0.55 | 1.75 |
| 2019-May | 0.0000001 | 0.11 | 0.24 | 0.51 | - | 0.78 |
| 2019-Jun | 0.0000001 | 0.1 | 0.04 | 0.15 | - | 0.77 |
| 2019-Jul | 0.0000001 | 0.03 | 0.0000001 | 1.3 | - | 0.08 |
| 2019-Aug | 0.0000001 | 0.0000001 | 0.28 | 1.03 | 0.03 | 0.61 |
| 2019-Sep | 0.11 | 0.13 | 0.36 | - | 0.06 | 0.38 |
| 2019-Oct | 0.68 | 1.49 | 0.66 | 2.43 | 0.24 | 0.42 |
| 2019-Nov | 0.32 | 8.03 | 0.34 | 4.82 | - | 0.37 |
| 2019-Dec | 0.15 | 6.54 | 0.08 | 3.49 | 0.53 | 0.24 |
| 2020-Jan | 0.16 | 8.95 | 0.09 | 4 | 0.53 | 0.46 |
| 2020-Feb | 0.14 | 9.87 | 0.22 | 3.87 | 0.51 | 0.58 |
| 2020-Mar | 0.24 | 12.32 | 0.59 | 5 | 0.71 | - |
| 2020-Apr | 0.21 | 3.05 | 0.54 | 0.55 | 0.41 | - |
| 2020-May | 0.0000001 | 0.0000001 | 0.0000001 | 0.45 | 0.19 | 0.24 |
| 2020-Jun | 0.03 | 0.14 | 0.0000001 | 0.56 | - | 0.33 |
| 2020-Jul | 0.17 | 0.64 | 0.14 | 0.55 | 0.05 | 7.39 |
| 2020-Aug | 0.0000001 | 0.0000001 | 0.13 | 1.43 | 0.0000001 | 0.77 |
| 2020-Sep | 0.13 | 0.89 | 0.27 | 1.88 | 0.11 | 2.02 |
| 2020-Oct | 1.04 | 4.02 | 0.33 | 4.2 | 0.26 | 1.21 |
| 2020-Nov | 0.17 | 5.08 | 0.14 | 3.93 | 0.37 | 0.73 |
| 2020-Dec | 0.1 | 5.93 | 0.09 | 3.19 | 0.4 | 0.67 |
| 2021-Jan | 0.12 | 9.37 | 0.06 | 3.67 | 0.48 | 0.97 |
| 2021-Feb | 0.22 | 9.97 | 0.07 | 4.03 | 0.54 | 0.56 |
| 2021-Mar | 0.29 | 9.93 | 0.28 | 3.34 | 0.52 | 0.85 |
| 2021-Apr | 0.27 | 4.45 | 0.18 | 1.41 | 0.36 | 4.06 |
| 2021-May | 0.02 | 0.05 | - | 0.74 | 0.1 | 0.83 |
| 2021-Jun | 0.02 | 0.04 | 0.12 | 0.35 | 0.03 | 2.6 |

**
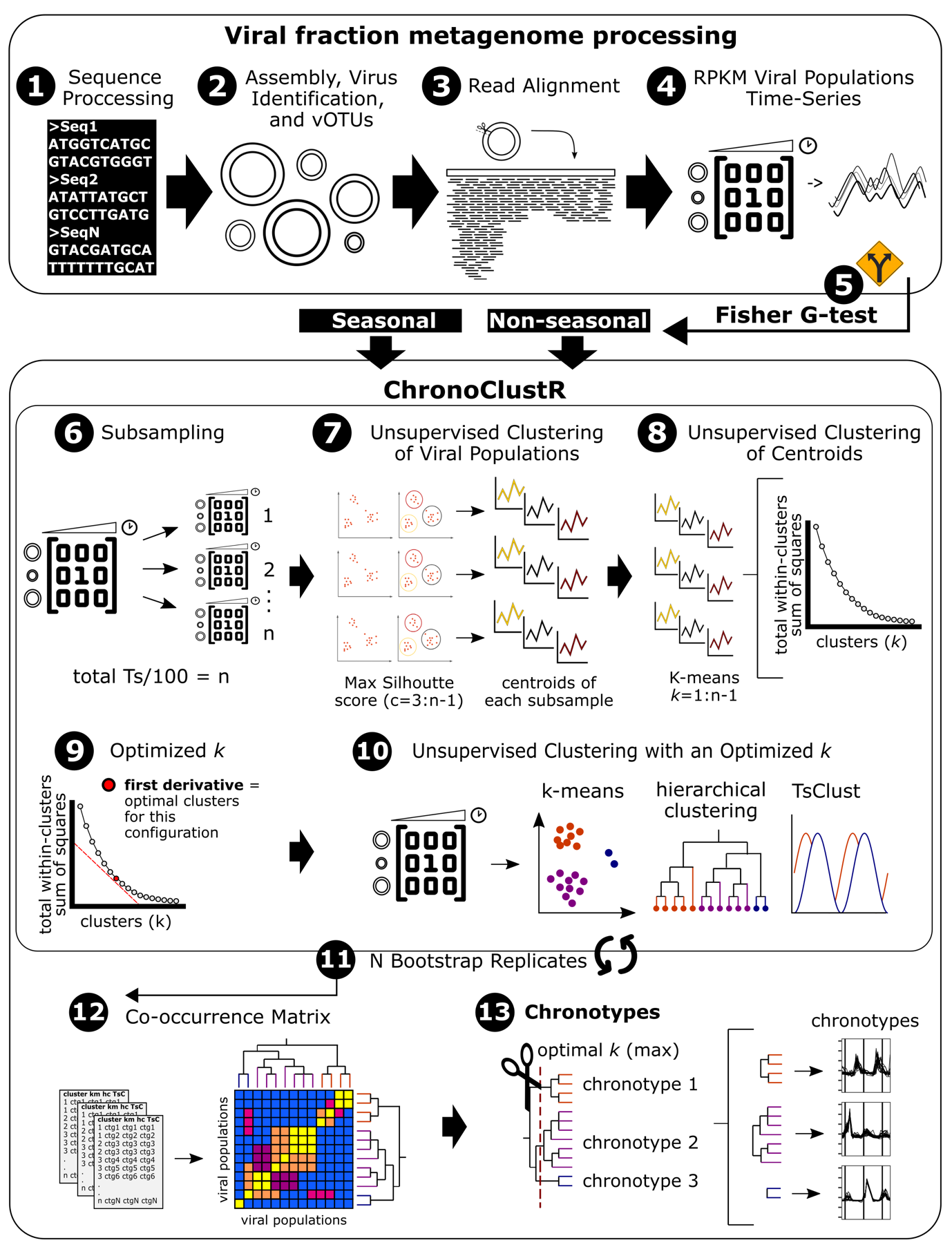
**

**Figure S1.- ChronoClustR:** a workflow to generate highly defined time-series chronotypes using an unsupervised machine learning algorithm. **(1-4)** Generate a vOTU RPKM matrix organised as a function of time: raw metagenomic datasets are quality filtered and decontaminated, assembled, classified as viral, dereplicated into vOTUs, and quality filtered. RPKMs were generated from the short-read alignment to the quality-filtered vOTUs. **(5)** Determine the seasonal and non-seasonal vOTU time-series. **(6-10)** Single round of unsupervised clustering for seasonal and non-seasonal vOTU time-series. The inputted collection of vOTUs time-series is subsetted into groups of 100 time-series (default) without replacement, each of these groups is clustered based on the Euclidean distance between them using the R package TSclust (selecting the *k* that outputs a maximum Silhouette score; being the *k*’s tested 3 to maximum number of elements -1), the generated centroids of all subgroups are subjected to a new clustering round using k-means for each of all the potential number of *k* clusters (minimum = 1 and maximum = total centroids -1). Based on the relationship between the number of clusters and the total within-clusters sum of squares, the program estimates an optimal *k* using the first derivative. The original dataset is reanalysed setting the optimal *k* generated in the previous steps. The workflow can do this clustering step using k-means, hierarchical clustering or TSclust. **(11-13)** Bootstrapping and chronotype definition. Steps 6 to 10 are bootstrapped (default = 100 replicates) to generate a matrix of co-occurence frequencies. This co-occurence matrix is used to generate a dendrogram which is then splitted into the maximum optimal *k* obtained through all bootstrap replicates (default). Each of the resulting groups will be considered a “chronotype”.

**
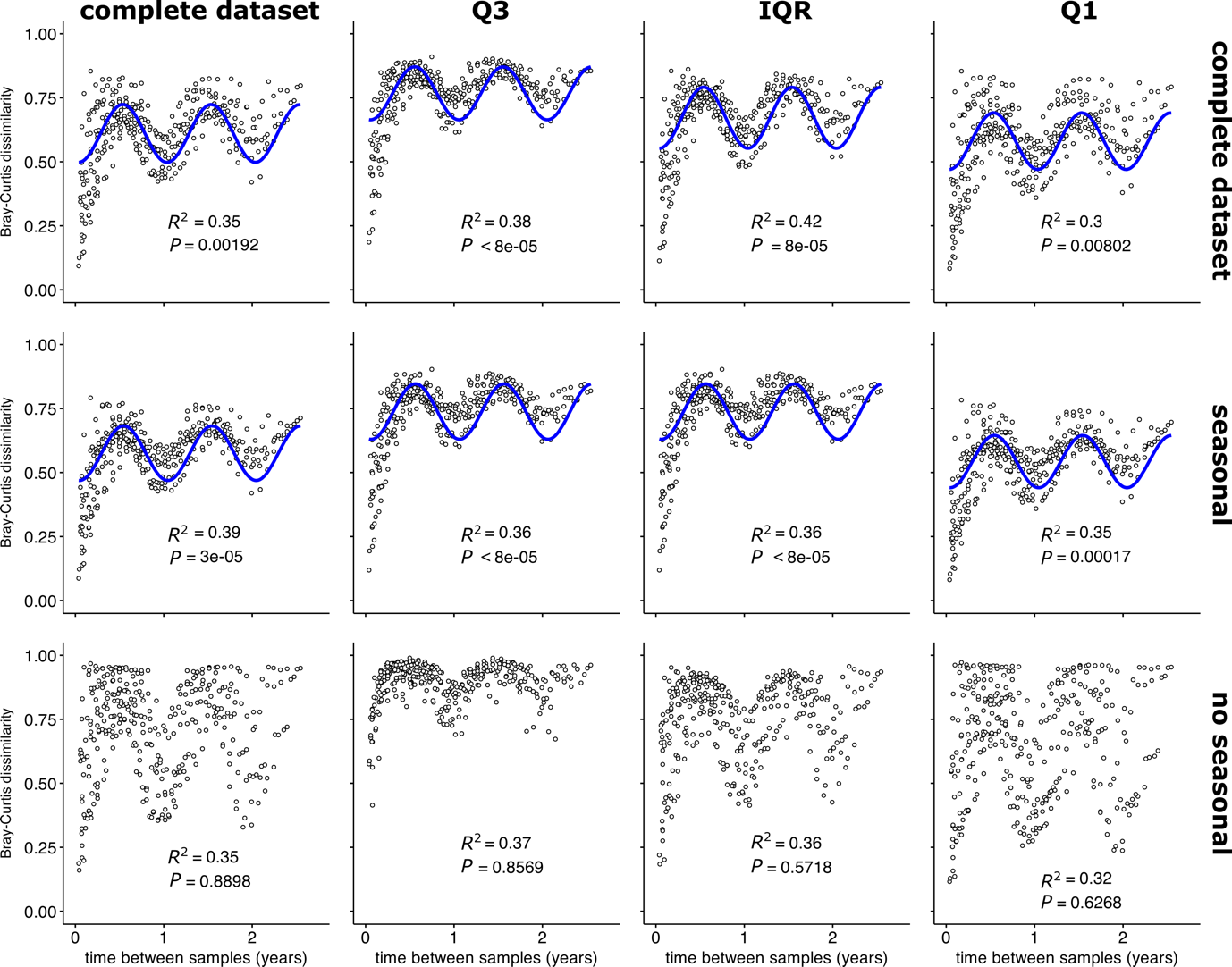
**

**Figure S2: Bray-Curtis dissimilarity time decay analysis of the high-quality virome composition in the Surface Western English Channel.** Pairwise dissimilarity in the viral community was estimated using the Reads Per Kilobase per Million mapped reads (RPKMs) values of 3,090 representative contigs over a period of more than two and a half years. The Bray-Curtis dissimilarities were averaged to establish a correlation with time distance (time gap between samples). A harmonic linear regression model was employed to identify significant seasonal trends in both the complete dataset and the analysed fractions. If the Bray-Curtis dissimilarity sinusoidal trend was statistically significant (p < 0.05), the linear regression was illustrated in blue. Columns are organized from left to right based on abundance, with the first column representing the complete dataset, followed by the third quartile, the interquartile range, and the first quartile of the RPKM distribution of the 3,090 representative contigs. Rows are organized from top to bottom, depicting the complete dataset (top), the seasonal fraction (middle), and the non-seasonal fraction (bottom) of the RPKM distribution of the 3,090 representative contigs.

**
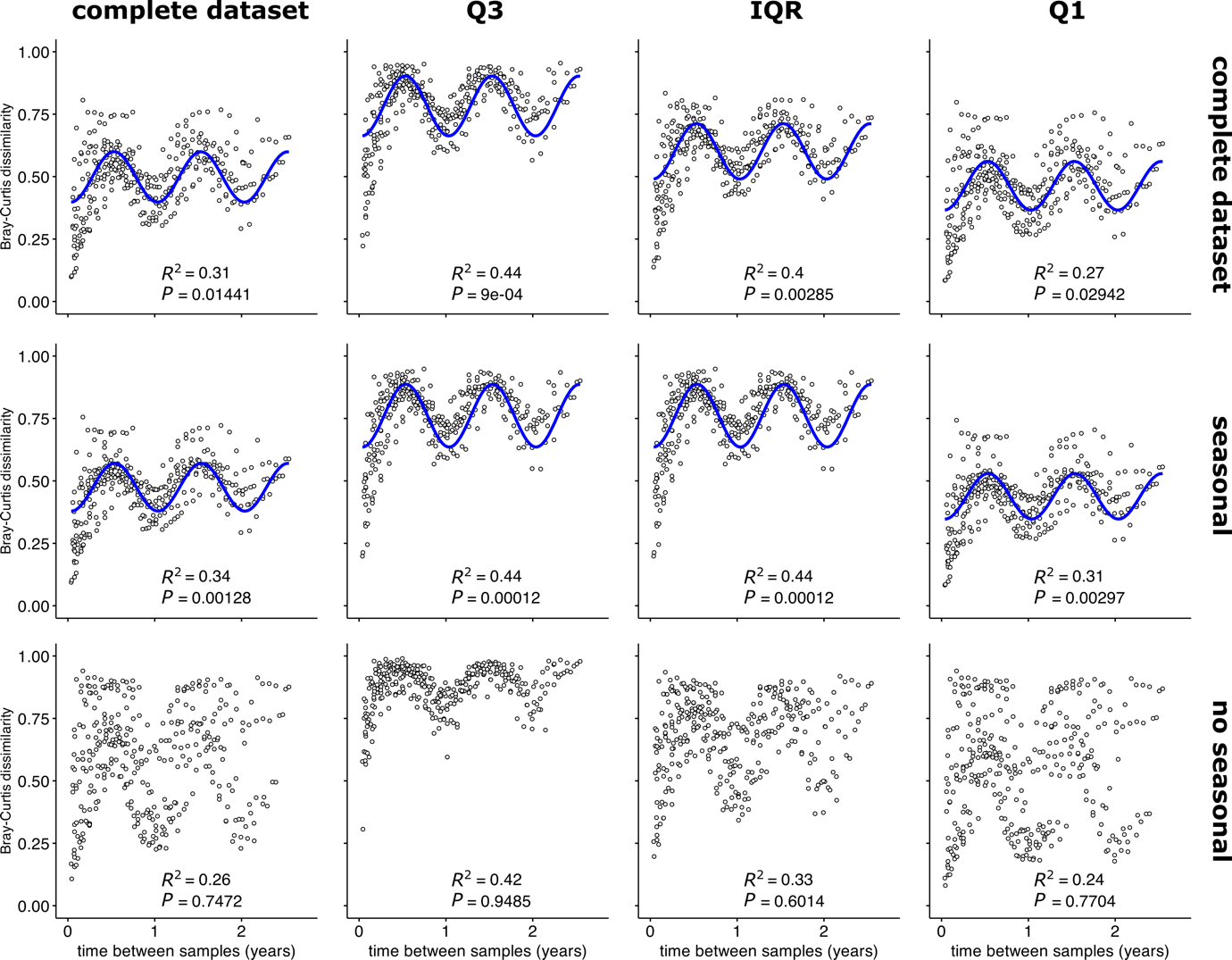
**

**Figure S3.- Bray-Curtis dissimilarity time decay analysis of the extended virome composition from the surface Western English Channel.** Pairwise viral community dissimilarity was estimated using the RPKMS values of the 26,851 population representative contigs over more than two years and a half. Identically to Figure S2, Bray-Curtis dissimilarities were averaged to generate a one-to-one correspondence between dissimilarity and time distance (time gap between samples). A harmonic linear regression model was used to determine seasonal trends in the complete dataset and the analysed fractions. If the Bray-Curtis dissimilarity sinusoidal trend was significant (p < 0.05), the linear regression was plotted in blue. Columns are organized from left to right in abundance terms, being the first the complete dataset and followed by the third quartile, the interquartile range and the first quartile of the RPKM distribution of the extended population representative contigs. Rows are organized from top to bottom: the complete dataset (top), the seasonal fraction (middle), and the non-seasonal fraction (bottom) of the RPKM distribution of the extended population representative contigs.


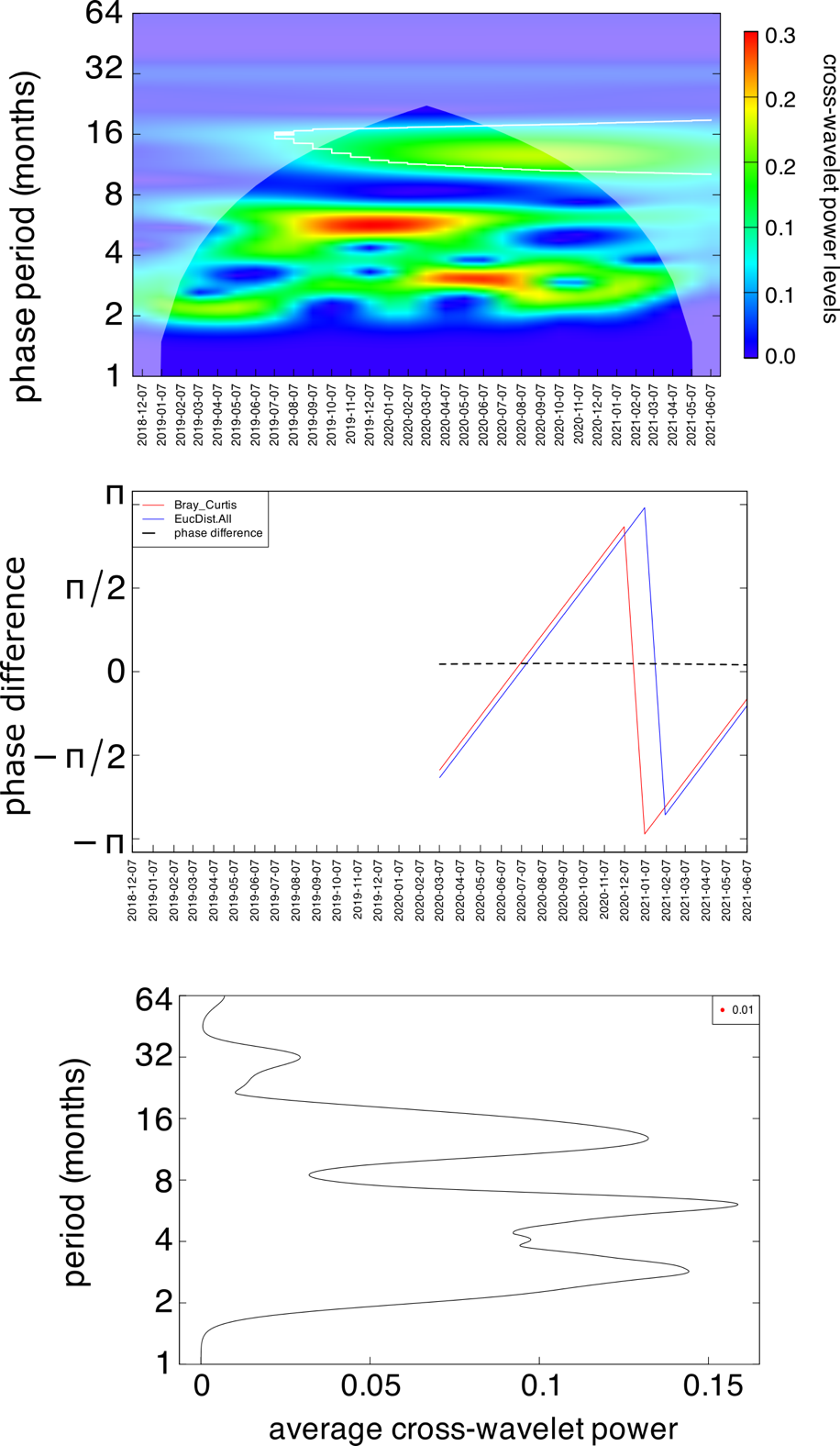


**Figure S4.- Wavelet coherence analysis between Bray-Curtis consecutive dissimilarities and Euclidean distances. (a)** Cross-wavelet power levels represented as a colour gradient. Periods with significant coherence (p < 0.05) are illustrated by a white line. **(b)** In phase representation of the two variables (red and blue) overlapped by the phase difference progression (dashed black line). **(c)** Average coherence for the significant in phase period (p < 0.01) is indicated as a red point over the specific significant periods. However, since no significant in-phase periods were detected, no red points are present along the line.


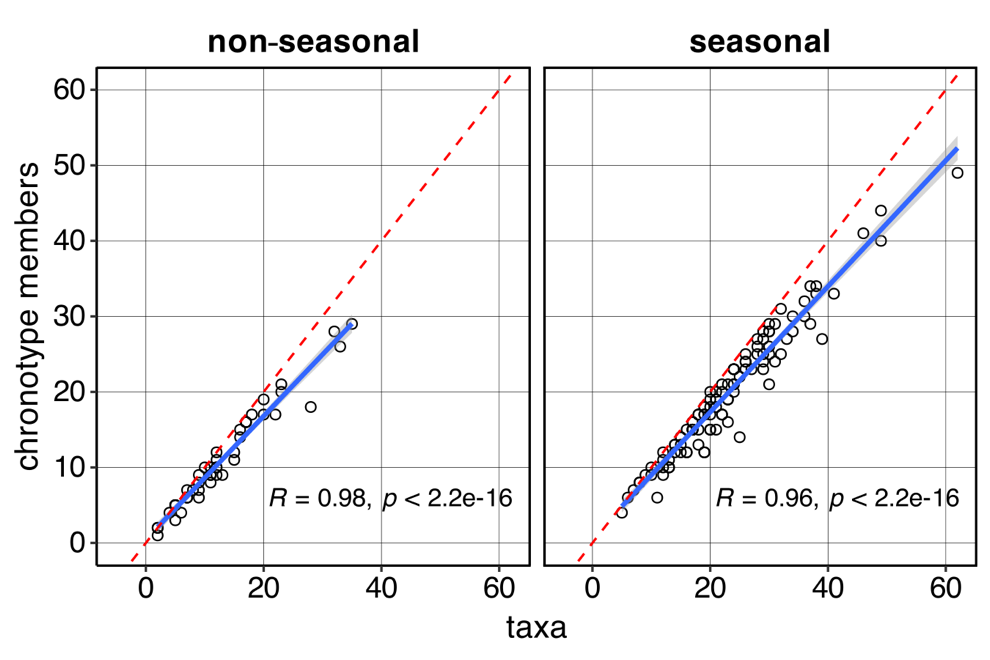


**Figure S5.- Viral Genus accumulation curves.** The taxonomic classification of the chronotype members were determined using gene-sharing profiles (vConTACT2 generated). Left panel shows the seasonal chronotypes and the right panel the non-seasonal. The dashed red line indicates a x=y linear relationship as reference. A significant positive linear correlation (spearman) exists between the number of vOTUs, and the number of genera represented in each chronotype.


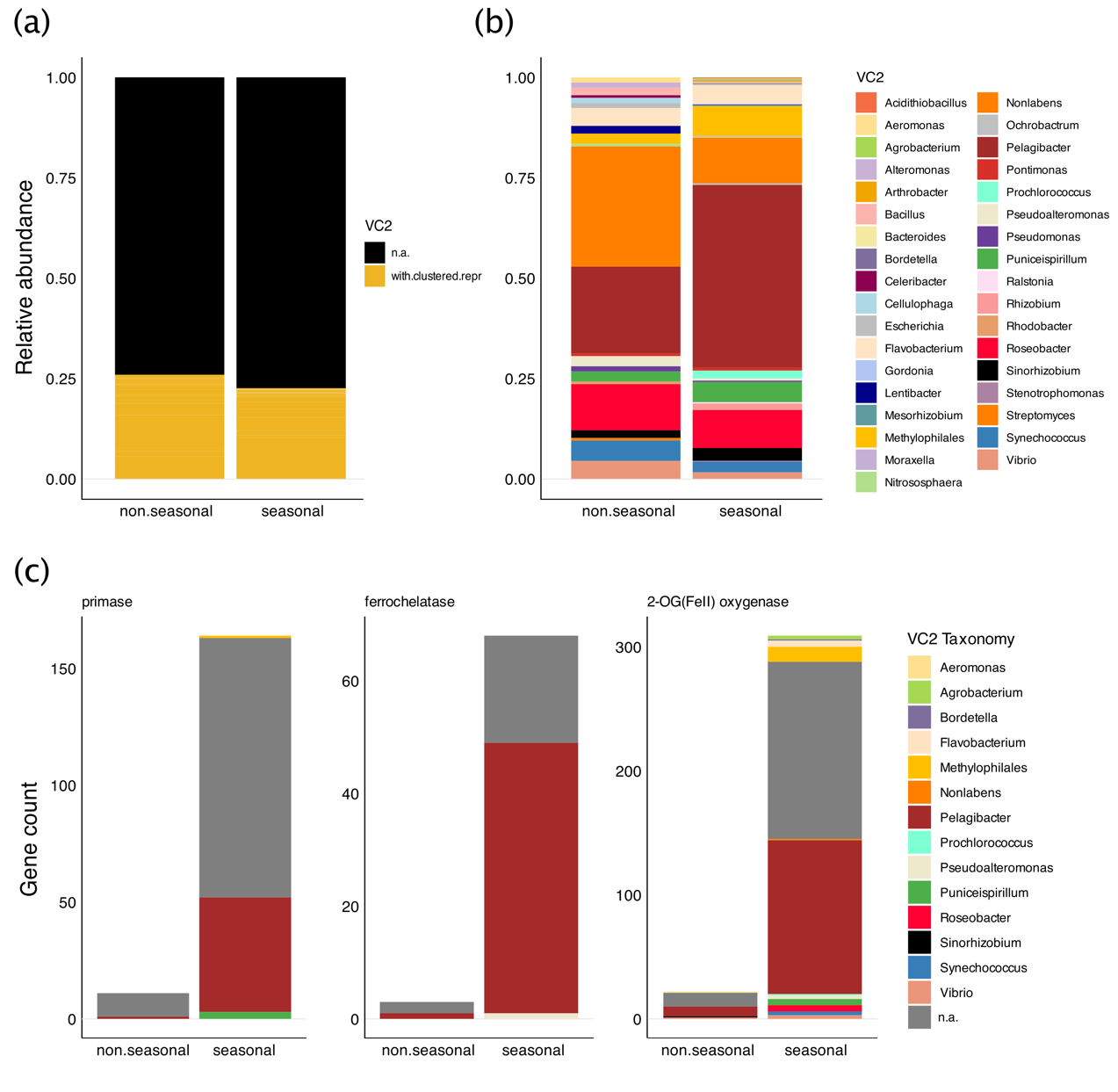


**Figure S6.- Metagenome assembled contigs taxonomic distribution and origin of seasonal enriched genes. (a)** Relative abundance of contigs with (gold) and without (black) a clustered representative in the INPHARED database. **(b)** Taxonomic relative abundance of the contigs with a clustered representative. **(c)** Stacked bar plots depicting the count of enriched genes in seasonal and non-seasonal chronotypes. Fractions are color-coded based on the taxonomic origin of the contig from which the genes were annotated.


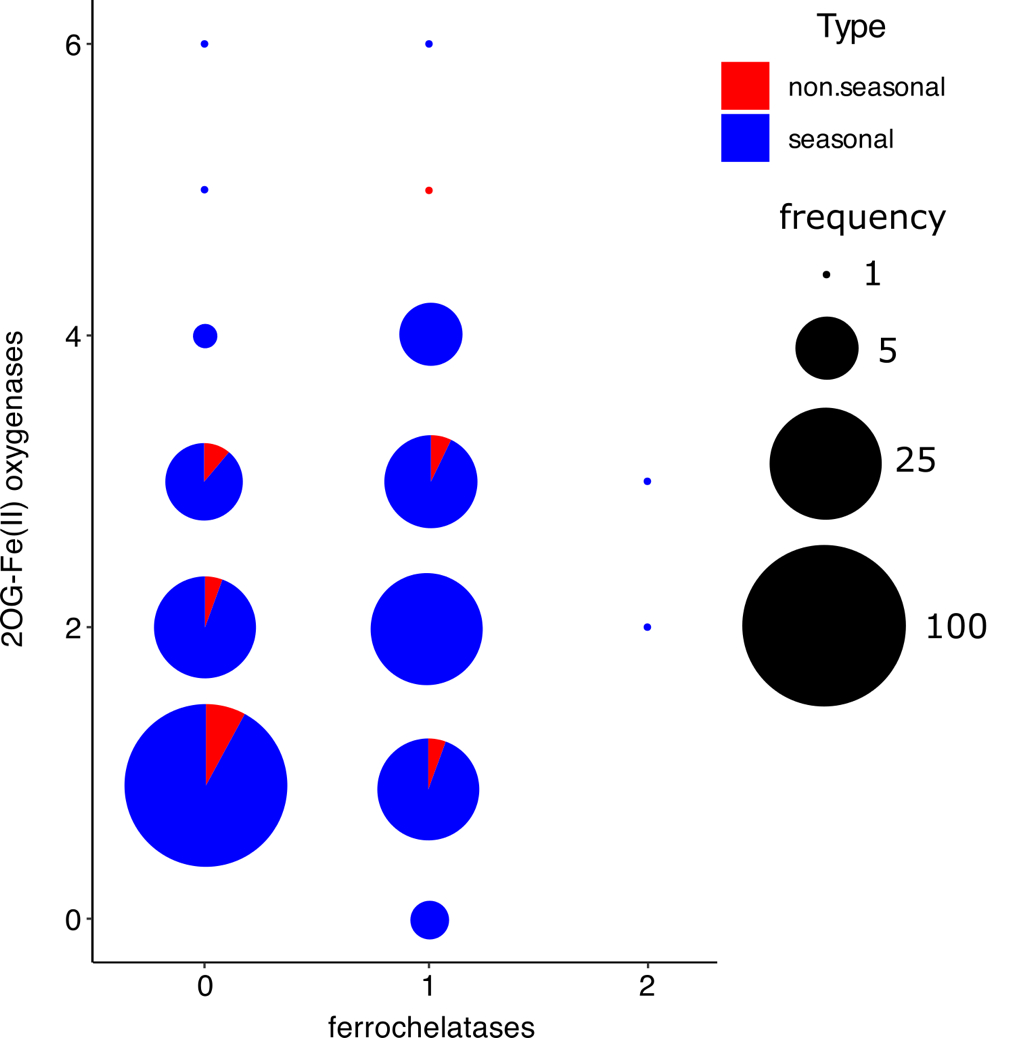


**Figure S7.- Co-occurrence of 2OG-Fe(II) oxygenase and ferrochelatase on the same population representative contig.** This plot represents the frequency of all pairwise combinations in the number of co-occurring 2OG-Fe(II) oxygenase and ferrochelatase coding sequences. Frequency is represented by the pie-chart size, while the percentage of seasonal and non-seasonal chronotypes that contribute to this frequency is represented by the color-coded fractions of the pie-charts.

**
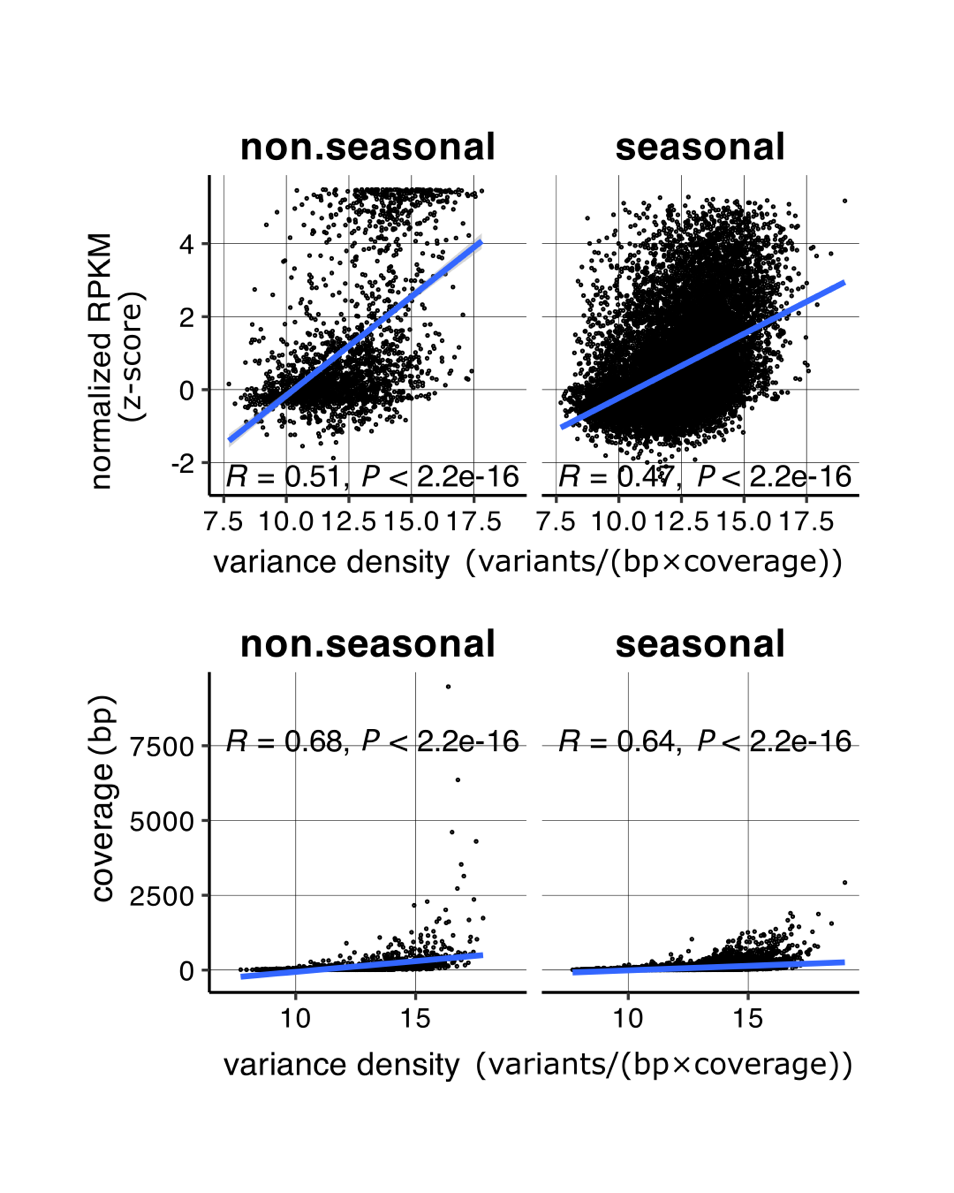
**

**Figure S8.- Correlation of variance density with normalized RPKMs and coverage of the high-quality vOTUs with a minimum coverage of 10X across 90% of the length in at least one sample.**

**
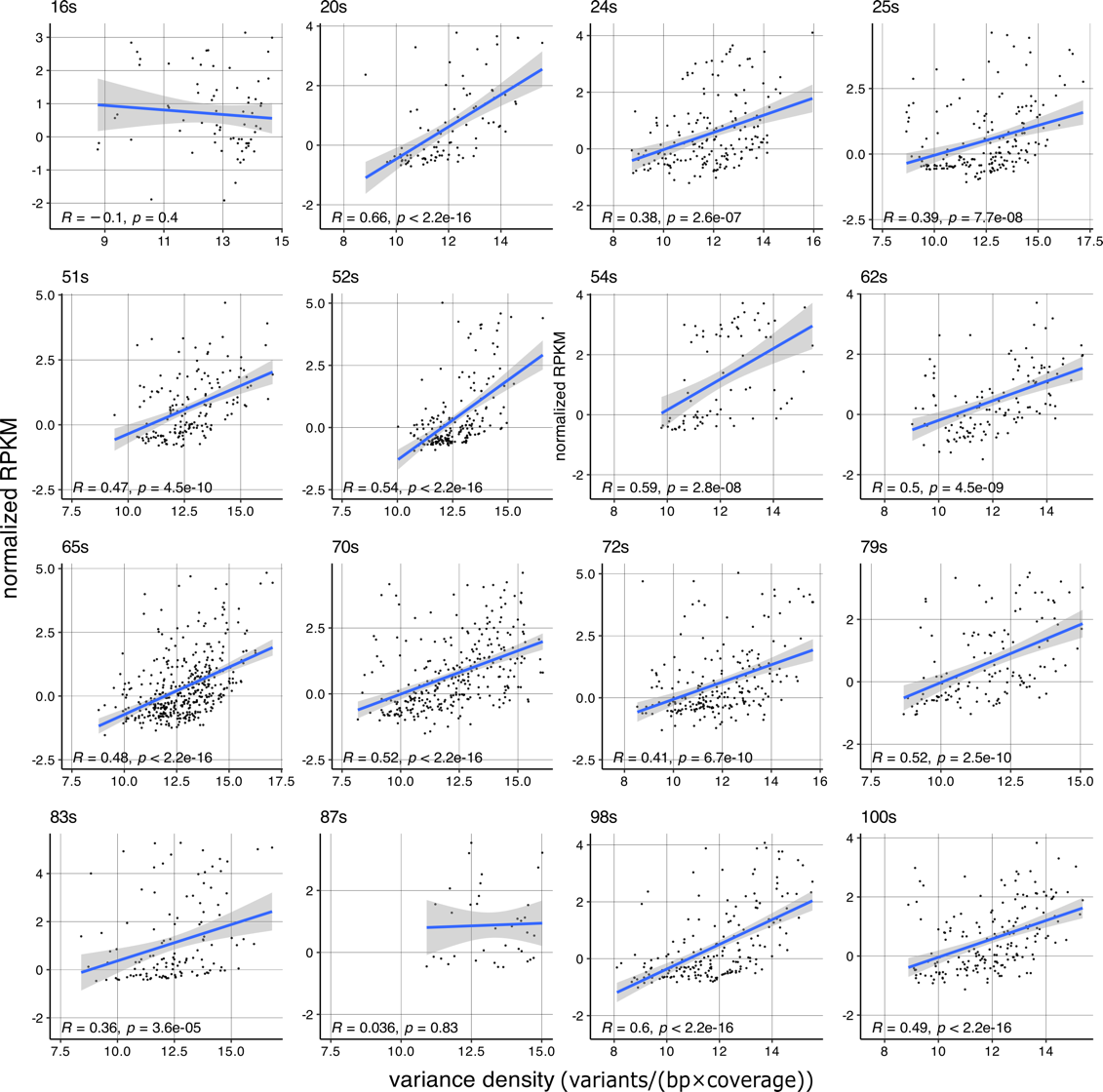
**

**Figure S9.- Correlation of variance density with normalized RPKMs of the vOTUs from the selected seasonal annual recurrent chronotypes.**

**
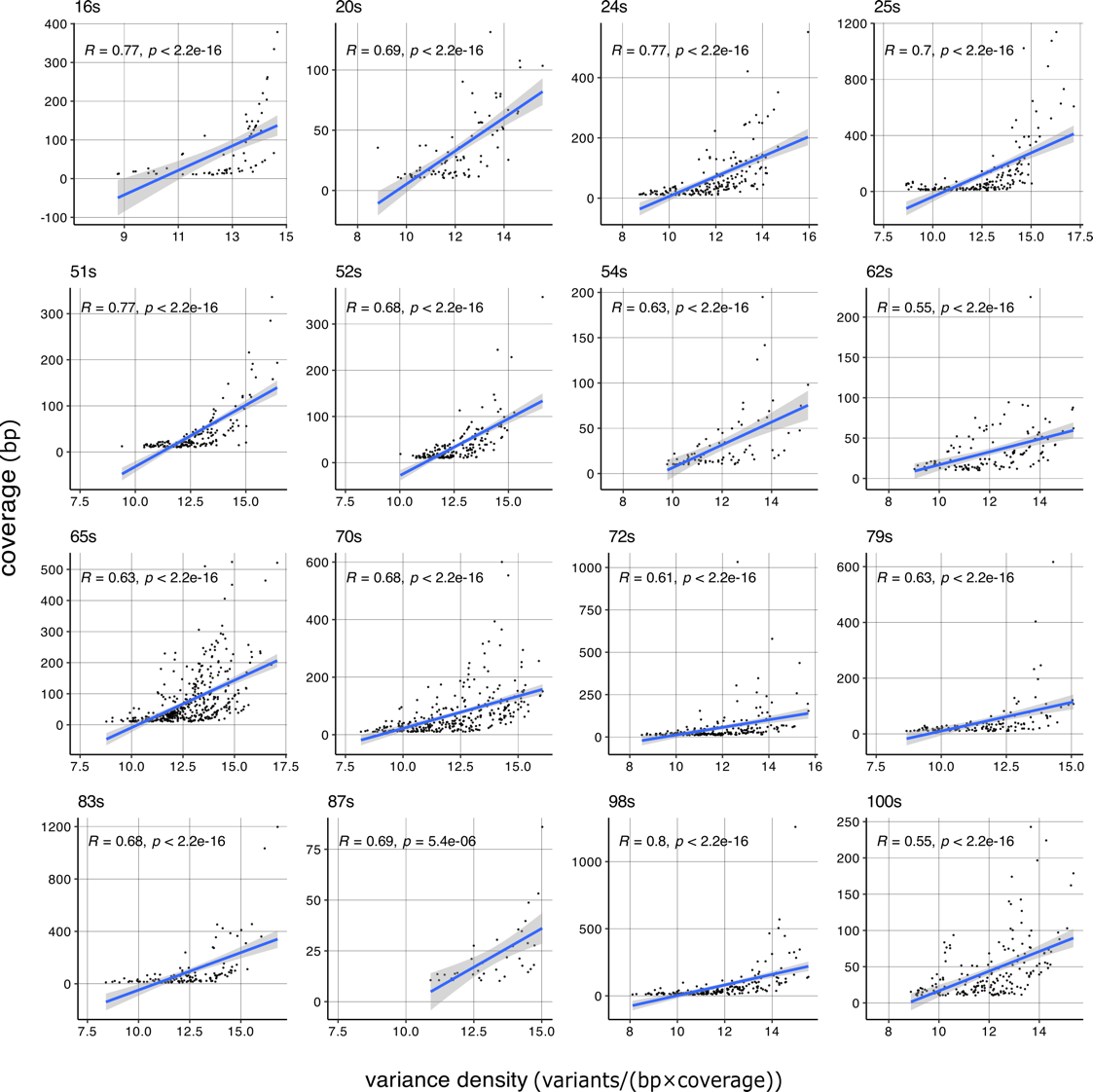
**

**Figure S10.- Correlation of variance density with coverage of the vOTUs from the selected seasonal annual recurrent chronotypes.**

**
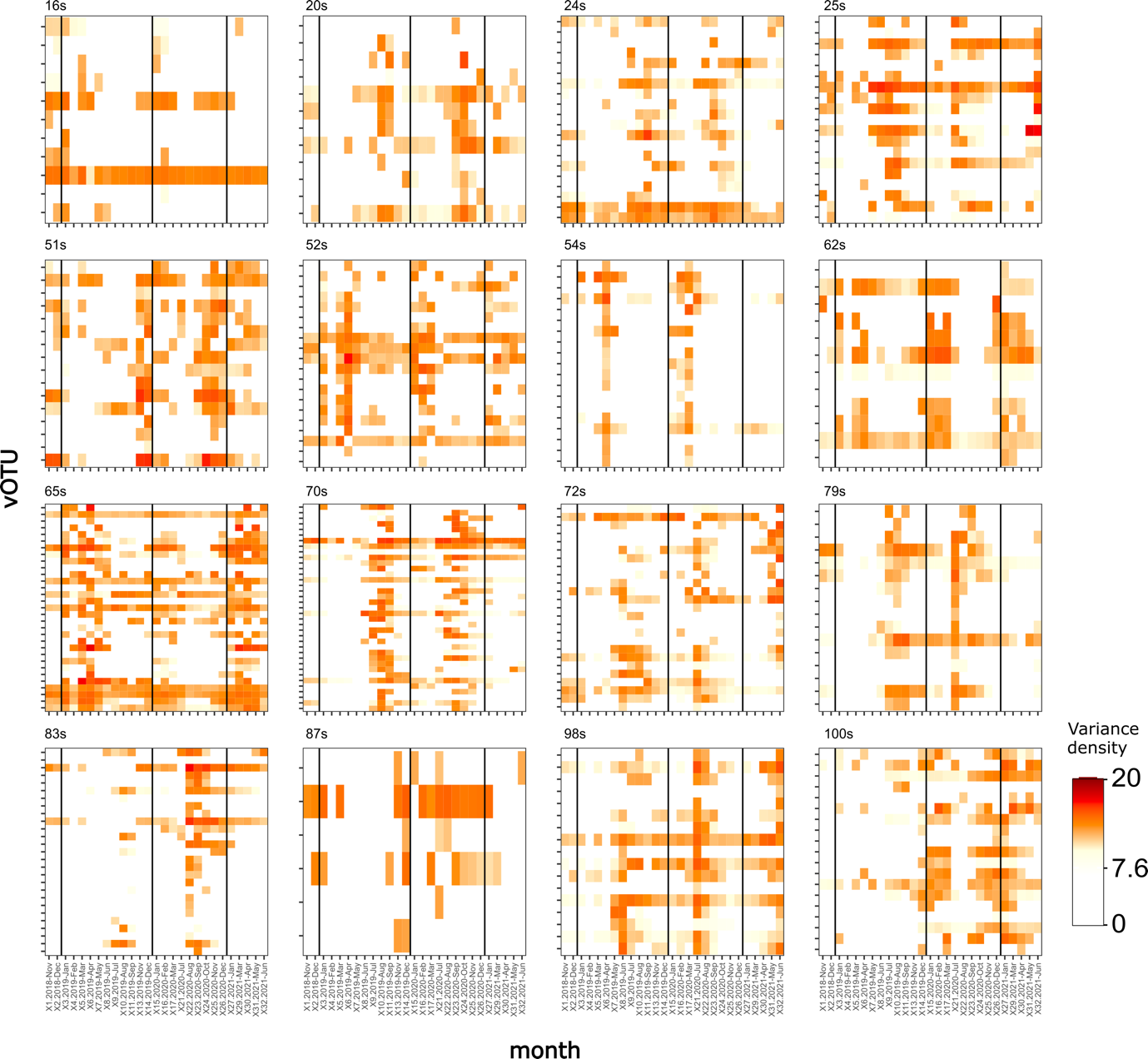
**

**Figure S11.- Variance density throughout the time series of the vOTUs from the selected seasonal annual recurrent chronotypes.** Each row represents a vOTU member of the chronotype (organized by panels). The variance density is chronologically ordered through the X-axis (left to right).

**
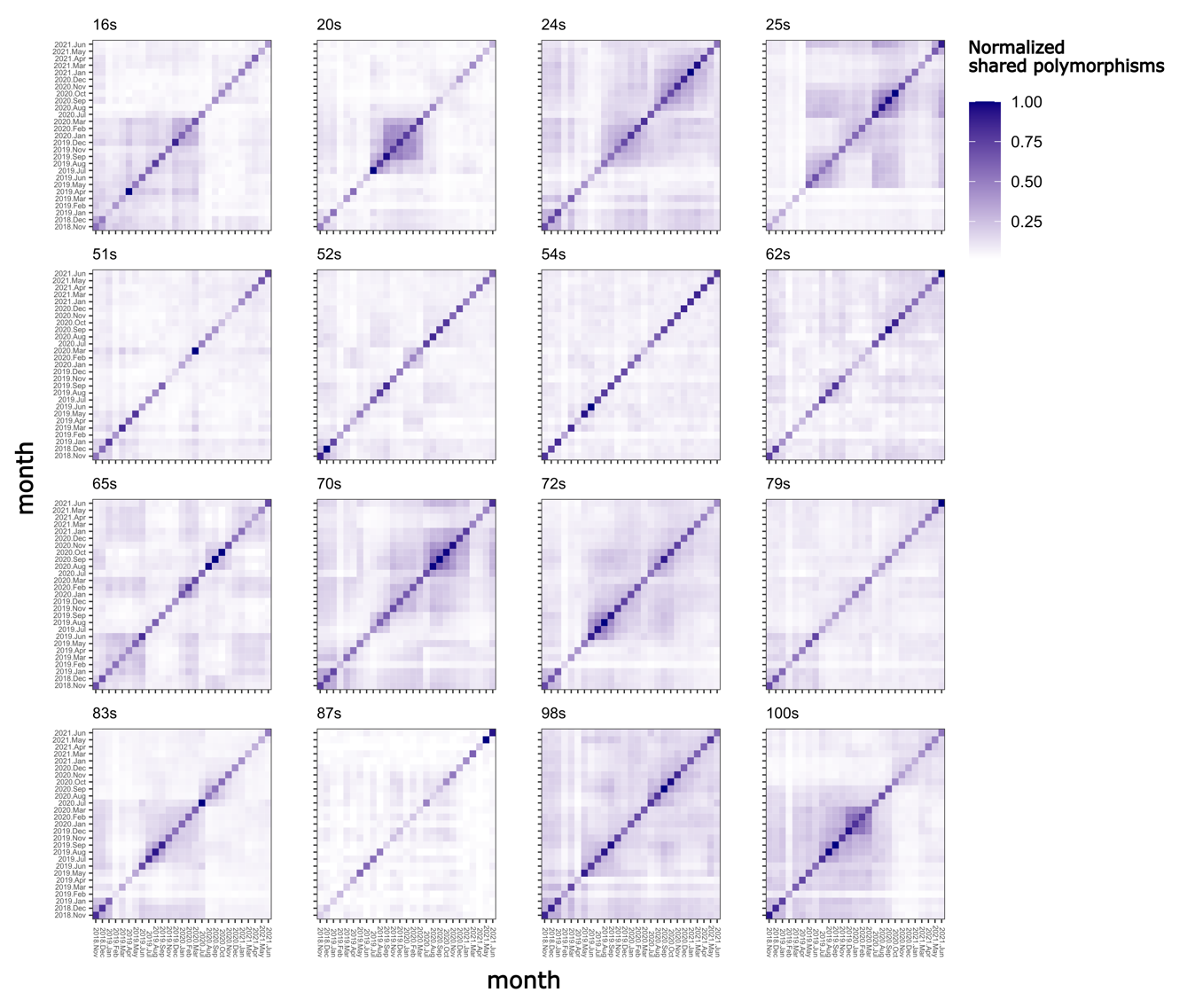
**

**Figure S12.- Monthly pairwise comparison of the normalized shared polymorphisms of 16 recurrent chronotypes.** For each chronotype we estimated the shared polymorphisms of all its member vOTUs in a pairwise comparison (all the potential combination of months). Each resulting chronotype matrix was normalized by setting the maximum value as 1 (feature scaling). Both the X-axis (left to right) and Y-axis (bottom to top) are ordered chronologically.


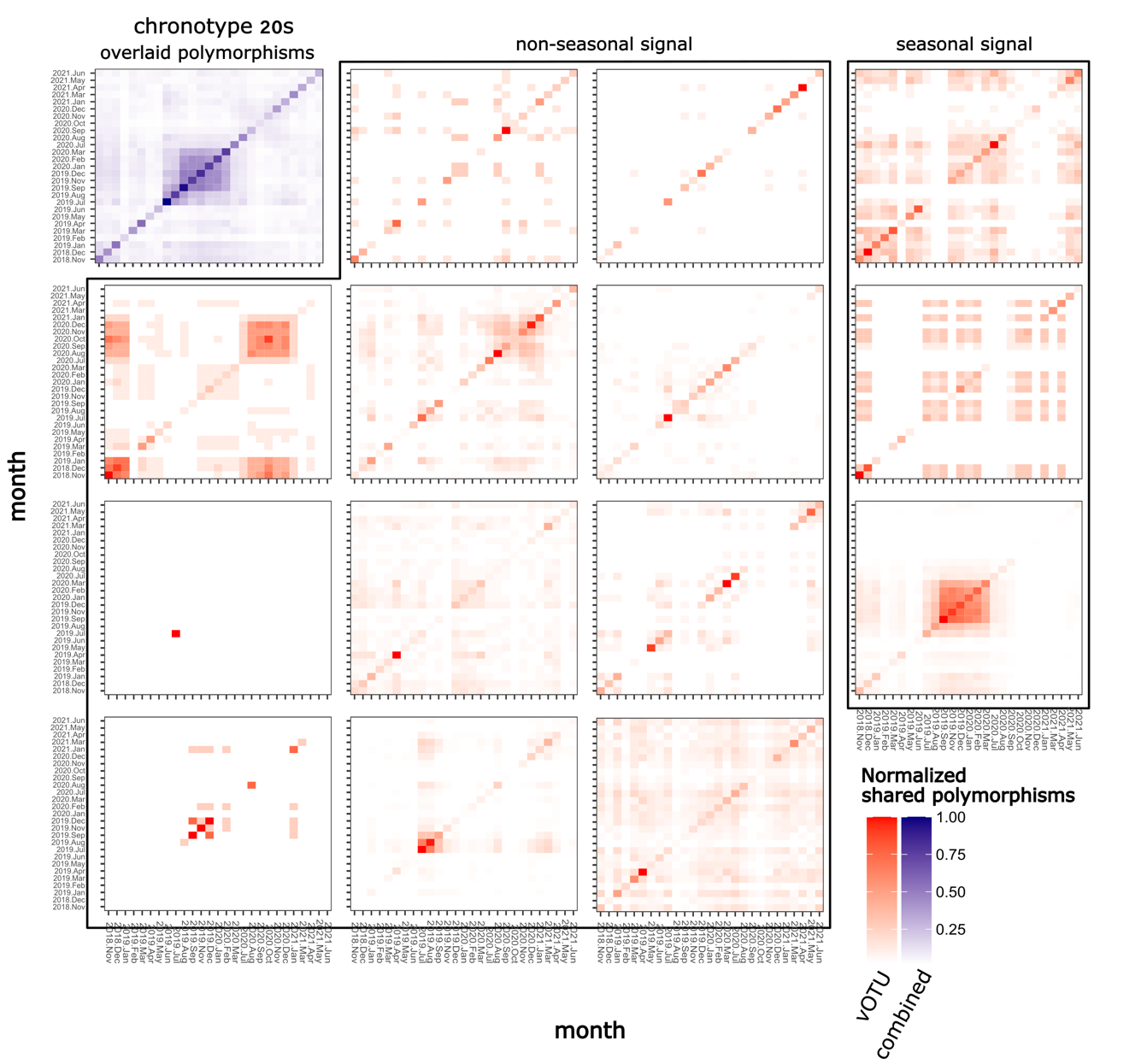


**Figure S13.- Recurrent chronotype 20s vOTUs display mostly a signal of shared polymorphisms consistent with monthly Red Queen dynamics along with a smaller fraction of an annual** recurrent signal of shared polymorphisms**.** vOTU members of a chronotype can have multiple patterns of shared polymorphisms. As in Fig. S11, the resulting matrices were normalized by setting the maximum value as 1 (feature scaling). The top right plot represents the merged chronotype profile of shared polymorphisms (combined analysis of all vOTUs in the chronotype, white-purple gradient as shown in Fig. S11). The rest of the panels (white-red gradient colours) are the individual vOTU profiles from where the top right panel was derived. vOTU panels were organized by placing those with no-seasonal signal on the left and the three vOTUs with a recurrent signal of shared polymorphisms on the right. Both the X-axis (left to right) and Y-axis (bottom to top) are ordered chronologically.
